# Online Peer Support for Long-Term Conditions: Protocol for a Feasibility Randomized Controlled Trial

**DOI:** 10.2196/71513

**Published:** 2025-07-23

**Authors:** Grace Lavelle, Hannah Grace Jones, Ewan Carr, Elly Aylwin-Foster, Vanessa Lawrence, Alan Simpson, Matthew Hotopf

**Affiliations:** 1 Department of Psychological Medicine Institute of Psychiatry, Psychology and Neuroscience King's College London London United Kingdom; 2 Department of Biostatistics & Health Informatics Institute of Psychiatry, Psychology and Neuroscience King's College London London United Kingdom; 3 Health Service and Population Research Institute of Psychiatry, Psychology and Neuroscience King's College London London United Kingdom; 4 Florence Nightingale Faculty of Nursing Midwifery & Palliative Care King's College London London United Kingdom

**Keywords:** chronic conditions, depression, long-term physical health conditions, mental well-being, online peer support, peer support, subthreshold depression

## Abstract

**Background:**

Over 30% of people in the United Kingdom are living with a long-term physical health condition. Early preventative peer support interventions could improve the lives and psychosocial well-being of people with long-term physical health conditions and reduce progression of any symptoms of low mood to more significant depression. In partnership with people with long-term conditions and industry stakeholders, we have co-designed an online peer support platform, CommonGround, to help people with long-term health conditions connect, support others, share experiences, and receive evidence-based information and advice on self-management.

**Objective:**

This feasibility randomized controlled trial will investigate whether the CommonGround platform is usable and acceptable for people with long-term physical health conditions experiencing mild depressive symptoms and whether conducting a future, larger confirmatory randomized controlled trial is feasible.

**Methods:**

A mixed methods, 2-arm, parallel-group, unblinded randomized controlled feasibility trial will be conducted nationally across the United Kingdom. Participants will include 150 adults (aged ≥18 years) who have access to the internet and are living with at least one long-term physical health condition and subthreshold depression (scoring 5-9 on the Patient Health Questionnaire–8). Following baseline assessments, eligible participants will be randomized to a coproduced online peer support and psychoeducation platform or a control condition where participants will receive fortnightly emails containing links to the National Health Service mental health web pages. Assessment measures will be collected at baseline and the midintervention (6 weeks) and postintervention (12 weeks) time points. A purposive sample of approximately 40 participants will be interviewed after the intervention to evaluate participant experiences and views on acceptability. The primary feasibility outcome is the number of participants recruited to the trial per week and in total via each recruitment route (as self-reported by participants).

**Results:**

Recruitment for the feasibility trial began on February 12, 2024. Quantitative data collection was completed by October 23, 2024, and qualitative data collection was completed by December 3, 2024.

**Conclusions:**

This trial will explore the acceptability and feasibility of our coproduced online peer support platform with embedded psychoeducational resources targeted for people living with long-term physical health conditions and subthreshold depression who are at risk of developing major depressive disorder. The findings will inform the future design of a larger randomized controlled trial exploring the platform’s clinical efficacy and cost-effectiveness.

**Trial Registration:**

ClinicalTrials.gov NCT06222346; https://clinicaltrials.gov/study/NCT06222346

**International Registered Report Identifier (IRRID):**

DERR1-10.2196/71513

## Introduction

### Background

In the United Kingdom, approximately 30% of the population are living with a long-term physical health condition [1], with almost 15% of the population in England living with ≥2 long-term conditions [2,3]. Mental health conditions are common among people living with long-term physical health conditions [4]. Almost 20% of patients attending outpatient hospital appointments in the United Kingdom meet the criteria for major depressive disorder [5], and a further 20% experience symptoms of low mood, known as subthreshold depressive disorder [6]. Subthreshold depressive disorder, defined as experiencing between 2 and 5 depressive symptoms for at least 2 weeks [5,7] and scoring 5 to 9 on the Patient Health Questionnaire, adversely impacts patients’ lives [8,9]. While psychological support may be offered to those reaching diagnostic thresholds for major depressive disorder (eg, National Health Service [NHS] talking therapies), there is little evidence of targeted psychological support for those living with long-term conditions who are experiencing subthreshold depressive disorder. This gap in mental health support for people with long-term conditions and subthreshold depressive disorder is a particularly pertinent issue given that the latter often precedes the development of major depressive disorder, which has downstream consequences for people with long-term conditions, their families, the NHS, and society [10,11]. Consultations with our patient and public involvement (PPI) group and the Mental Health Foundation’s Living Better focus group study [12] both identified the need for greater research attention into supporting the mental health of people living with long-term conditions. Therefore, research into the development of low-cost and accessible early interventions for those with long-term conditions and subthreshold depressive disorder is warranted.

Online peer support interventions have the potential to offer inexpensive and scalable solutions where people with similar experiences interact and exchange emotional, informational, and appraisal support [13-15]. In particular, the online setting allows the support to be constantly available and overcomes practical barriers to physically accessing in-person support groups [16]. Following COVID-19, patients are also more comfortable with remote technologies in health care [17,18], with patients reporting that online peer support can also provide reciprocal social support [19-22] and access to the experiential knowledge [15,19,21] that face-to-face peer support provides. Numerous randomized controlled trials (RCTs) document the benefits of in-person peer support for decreasing depressive symptoms, increasing self-efficacy, and improving physical health outcomes [23,24]. In contrast, the number of high-quality RCTs exploring the safety and effectiveness of online peer support among people with long-term conditions is limited, and there are inconsistencies in the outcome measures administered. However, a recent scoping review found evidence of significant improvements in social participation, self-efficacy, and health-directed activity alongside reduced emotional distress due to engaging with online peer support [14]. Other studies on specific conditions such as cancer [25] have documented small to moderate reductions in depression, providing early evidence for the benefits of online peer support. Typically, online peer support communities are condition specific, overlooking the opportunity for people living with *any* long-term condition to reciprocate support based on *their shared experience of living with a long-term condition*. Furthermore, while it is suggested that patients may respond better to interventions addressing the mind-body link [26], to our knowledge, there are currently no online peer support communities specifically tailored to supporting patients’ psychological well-being within the context of their physical health.

Participatory action research has been identified as a cornerstone in building successful online communities tailored to the wants and needs of people with long-term conditions [27]. Indeed, the United Kingdom’s Five Year Forward View for Mental Health recommended expanding the provision of coproduced peer support interventions [28]. These views are echoed in what people with lived experience of long-term conditions have told us in our prior work: that online peer support interventions must be carefully co-designed, safe, and nonprescriptive and not feel commercialized or motivated by profit if they are to be liked and used by patients [29]. Therefore, our ultimate goal is to cocreate an online peer support platform that intervenes early to prevent comorbid major depressive disorder in people living with long-term conditions. Our collaboration with people with lived experience of mental-physical comorbidities is central to our work through our PPI groups and coapplicant with lived experience alongside clinicians, academics, and industry partners. We have collaborated for 18 months to iteratively co-design an online peer support platform with embedded psychoeducational resources called CommonGround. To inform the wider adoption of our intervention within health services, the next step is to conduct a feasibility RCT (fRCT) to evaluate the acceptability of the intervention and the feasibility of a future confirmatory trial to assess the platform’s clinical effectiveness.

### Objectives

The primary aim of this trial is to assess the feasibility and acceptability of our coproduced online peer support platform (CommonGround) for people living with long-term conditions and subthreshold depressive disorder and inform the design of and the feasibility of conducting a larger confirmatory trial to assess clinical efficacy and cost-effectiveness. Our specific objectives are to (1) estimate recruitment and retention rates; (2) determine participants’ perspectives on the acceptability and usability of the platform and the acceptability of the trial procedures; and (3) determine the parameters needed to estimate the sample size for a future confirmatory RCT, including the incidence of major depressive disorder and SDs of the Patient Health Questionnaire–8 (PHQ-8) [30] and Warwick-Edinburgh Mental Well-Being Scale [31] in this population and preliminary estimates of treatment effects for clinical outcomes.

## Methods

### Design

A mixed methods, 2-arm, parallel-group, unblinded randomized controlled feasibility trial with a nested qualitative study will be conducted. We adopted a mixed methods approach to generate comprehensive and contextually grounded insights into the feasibility and acceptability of the intervention and trial design [32,33]. Quantitative data will support the assessment of key feasibility criteria for progression to a future efficacy trial. This includes evaluating recruitment, retention, and completion rates as well as responses to outcome measures.

Qualitative data will explore the acceptability and feasibility of both the intervention and trial procedures from the perspectives of participants and the moderation team. In particular, these data will identify views on the usability and acceptability of the CommonGround platform and opportunities for intervention optimization. Quantitative measures related to intervention acceptability, such as usability and sense of community, will be analyzed alongside interview data to provide a richer, integrated understanding of user experience. Data analysis will be collaborative, with qualitative and quantitative researchers collaborating to triangulate findings and inform future optimization and testing of the intervention and trial design.

### Study Setting

This trial will be conducted remotely, with all assessments, interviews, and treatments delivered online. All participants will be recruited in the United Kingdom by King’s College London (KCL).

### PPI Aspect

This trial builds on prior work involving the coproduction of the online peer support platform rooted in PPI. The platform prototype was co-designed based on initial focus group insights on the psychosocial needs of people with long-term health conditions [29] and via a series of participatory design panel workshops. The participatory design panel workshops helped develop and translate a theory of change for the intervention’s mechanisms into practical intervention components (eg, features that the peer support forum should have). The prototype then underwent subsequent content reviews by our research advisory group (RAG). The full details on the co-design and usability testing of the CommonGround platform will be published elsewhere.

For this trial, our RAG continued to provide input into the study procedures and documents and co-designed the adverse event (AE) schedule. From the initial development work, our RAG expanded to involve 10 members (n=6, 60% female and n=4, 40% male aged between 25 and 73 years) living with various long-term physical health conditions. In total, 60% (6/10) of the members were living with multiple long-term conditions, and 50% (5/10) had comorbid mental health diagnoses. During the trial, the RAG will continue to meet regularly to shape the development of the coding framework for the qualitative data analysis and provide feedback on the interpretation and dissemination of results. In addition, one of the coauthors (EA-F) has lived experience of multiple long-term conditions and expertise in health communications and has been a core member of our research team, helping ensure that the conduct of this study is firmly grounded in the experience of people living with long-term conditions.

### Participants and Recruitment

#### Inclusion Criteria

Participants must be living with probable subthreshold depressive disorder (scoring 5-9 on the PHQ-8); be currently living with one or more long-term physical health conditions; be aged ≥18 years; have access to the internet (via phone, desktop, or laptop at any location, including at home, at the workplace, or free Wi-Fi in public spaces); have sufficient English proficiency to engage with the platform (self-assessed during screening); and have the ability to provide informed consent (ie, no known circumstances or conditions that may affect the capacity to consent).

Although there are no restrictions on the type of long-term condition (refer to Multimedia Appendix 1 for a list of common long-term condition options presented to interested participants), the long-term condition must be a diagnosed physical medical condition with a duration of ≥6 months that demonstrates recurrence or deterioration and is typically associated with a poor prognosis [34].

#### Exclusion Criteria

We will exclude participants who have ever received a diagnosis of dementia or a clinical diagnosis of severe mental illness of bipolar disorder, psychosis, posttraumatic stress disorder (PTSD), or schizophrenia. CommonGround has been specifically designed to prevent the onset of depressive episodes in those with subthreshold symptoms of depression and, therefore, may not be appropriate support for other groups of people. Individuals with established mental disorders and dementia will be excluded because CommonGround is not designed to support their mental health needs or able to support people with cognitive deficits who may find the platform problematic. All exclusion criteria are self-reported during screening.

### Recruitment

#### Overview

In total, 5 potential recruitment routes are planned to minimize the time needed to recruit the required sample size.

Route 1 comprises consent to contact patient databases (eg, the BioResource database and Lambeth NHS Talking Therapies Service). The research team or gatekeepers from these organizations will contact individuals to share the online participant information sheet.

Route 2 comprises outpatient clinics at King’s College Hospital NHS Foundation Trust and Guy’s and St Thomas’ NHS Foundation Trust that have used the Integrating Mental and Physical Healthcare: Research Training and Services (IMPARTS) platform [35]. Clinicians in IMPARTS clinics will share information sheets with potentially suitable patients, and flyers will be displayed in waiting rooms to invite interested participants to contact the research team directly or access the online participant information sheet.

In route 3, outpatient clinics at King’s College Hospital NHS Foundation Trust and Guy’s and St Thomas’ NHS Foundation Trust where IMPARTS has not been implemented will be approached to facilitate recruitment. Other primary or secondary care settings may also be approached (eg, general practices and other NHS Trusts with specialized clinics for long-term conditions). Clinicians will offer patients information sheets and display flyers in waiting rooms, inviting interested participants to contact the research team directly or access the online participant information sheet.

In route 4, public advertisements will be used throughout the social media accounts of KCL, King’s Health Partners [36], and the research team (eg, X [formerly known as Twitter] and Facebook); through internal and external KCL communications and bulletins (eg, the disability team); and through flyers displayed around KCL campuses and other suitable public areas (where permission is granted). Flyers and advertisements will also be disseminated to the public through charity organizations.

Route 5 comprises word of mouth and snowballing. We will invite screened individuals or those who have already heard about the study to share the study information with their peers.

#### Sample Size

Sample sizes ranging between 24 and 50 are commonly recommended for feasibility studies, with Teare et al [37] recommending 70 participants for a 2-arm RCT (35 per arm). However, larger samples are typically required for online, group-based peer support interventions to gain traction as this form of peer support depends upon interactions among a large number of participants to maximize the volume of discussions and interactions that develop a sense of community. Therefore, to ensure an adequate group size for the platform, we aim to recruit 150 participants and randomize them 2:1 into intervention and control arms, respectively. Recruitment is expected to last 10 weeks. Recruiting 150 participants will allow us to estimate an expected recruitment rate (primary feasibility outcome: number recruited to the trial per week) of 15 participants per week (95% CI 12.7-17.6) based on an exact Poisson test. Given the aims of this feasibility trial, no power calculations for clinical outcomes were conducted.

#### Participant Timeline and Study Procedures

**Figure 1:**
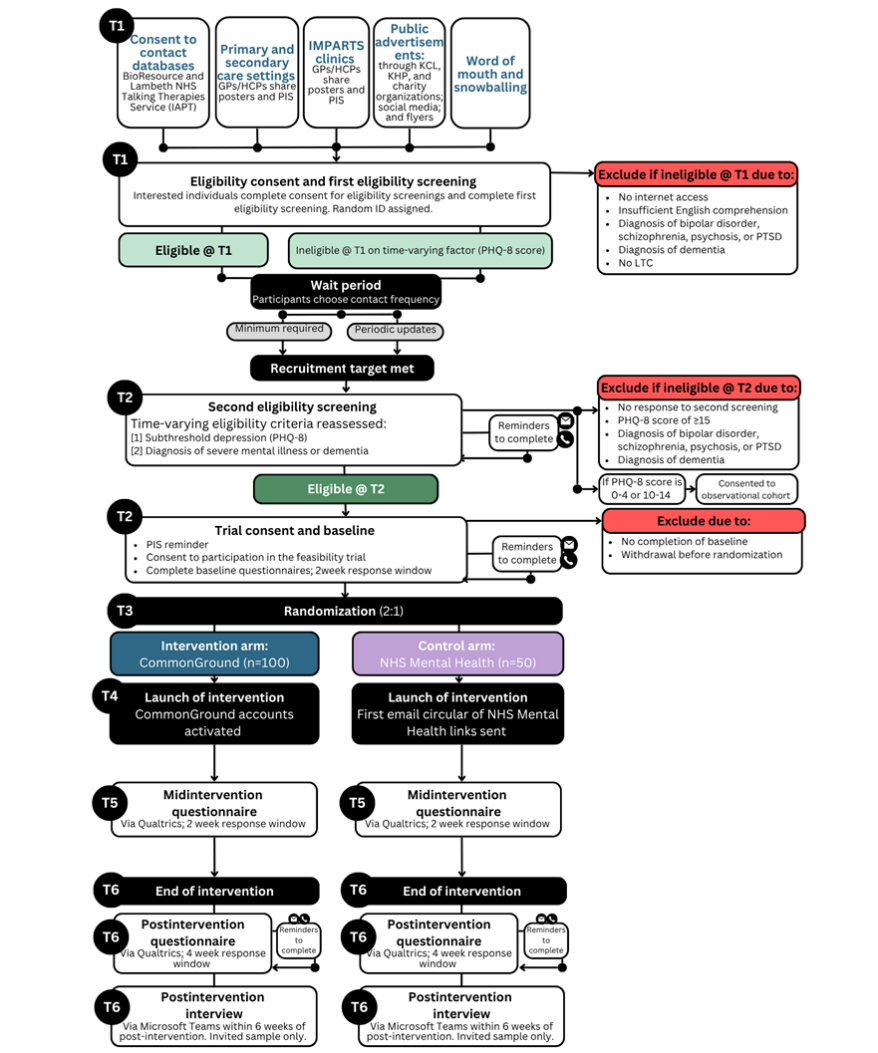
Flowchart showing the participant timeline from recruitment (T1) to the end of data collection (T6). GP: general practitioner; HCP: health care professional; IAPT: Improving Access to Psychological Therapies; IMPARTS: Integrating Mental and Physical Healthcare: Research Training and Services; KCL: King’s College London; KHP: King’s Health Partners; LTC: long-term physical health condition; NHS: National Health Service; PHQ-8: Patient Health Questionnaire–8; PIS: participant information sheet; PTSD: posttraumatic stress disorder.

The flow of participants through the trial is summarized as follows (and illustrated in Figure 1): (1) recruitment (T_1_); (2) consent to take part in eligibility screenings (T_1_); (3) first eligibility screening (T_1_); (4) wait period; (5) second eligibility screening, consent to trial, and baseline assessment (T_2_); (6) randomization (T_3_); (7) intervention period (T_4_); (8) midintervention assessment (T_5_); and (9) follow-up assessment and qualitative interviews (T_6_).

All interested individuals will be directed to the trial URL or QR code on the recruitment materials to access the online participant information sheet hosted on Qualtrics (Qualtrics International Inc; T_1_). Participants will have the opportunity to contact the research team with any questions. Those who read the study information and decline to consent will be asked to anonymously indicate their reasons for declining to identify potential recruitment barriers. Those interested will consent to the eligibility screenings and complete the first screening (T_1_). Participants who are living outside the United Kingdom, are aged ≤17 years, do not have access to the internet, do not have a long-term physical health condition, do not have sufficient English proficiency, do not have the capacity to consent, or are living with a diagnosis of dementia or a severe mental health condition (bipolar disorder, psychosis, PTSD, or schizophrenia) will be informed that they are not eligible for the trial. All ineligible participants will be provided with information on available support and invited to contact the research team for further information.

Participants meeting all the inclusion criteria will be invited to enter the wait period. Participants ineligible at T_1_ *based solely on their PHQ-8 score* will also be invited to enter the wait period as PHQ-8 scores and, therefore, eligibility may change over time. The wait period is necessary so that all participants randomized to the peer support platform gain access simultaneously, maximizing the opportunity for discussion and the formation of a community. The wait period is a trial design feature and would not be necessary if CommonGround were implemented in the health care system as people with long-term health conditions could immediately enroll and gain access to the preexisting community. During the wait period, participants can opt in to receive recruitment updates via email and can access a live URL summarizing recruitment progress via the study website. The wait period will finish when the recruitment target is met (N=150). Participants will then be invited to complete the second, shorter screening via individually generated Qualtrics links to assess their eligibility on two time-varying inclusion and exclusion criteria (T_2_): (1) meeting the criteria for probable subthreshold depressive disorder (scoring 5-9 on the PHQ-8) and (2) no new diagnosis received during the wait period of bipolar disorder, psychosis, PTSD, schizophrenia, or dementia.

All individuals found to be eligible at the second screening will be invited to consent to the fRCT and complete the baseline assessment (T_2_). Participants have 2 weeks to complete consent and baseline assessments via their individually generated Qualtrics link. The research team will contact participants who do not respond to the rescreening invitation a maximum of 3 times via email, with a follow-up phone call if necessary. Those individuals who are found ineligible for the fRCT solely due to their PHQ-8 score (ie, they satisfy all other inclusion criteria) and have PHQ-8 scores of ≤4 or within the range of 10 to 14 will be offered the opportunity to consent to a community engagement observational cohort. Participants in the community engagement observational cohort will be onboarded onto CommonGround alongside trial participants and engage with the platform as they wish during the 3-month intervention period. The community engagement cohort will increase the size of the peer community, potentially facilitating the intervention to gain traction. The protocol for the engagement cohort is available in Multimedia Appendix 2 and is not described further in this paper.

Following the baseline assessment (T_3_), participants will be randomly assigned (2:1) to the CommonGround platform or the control group. The intervention period will begin on the trial launch date (T_4_). At T_4,_ the control group will receive the first email sharing the NHS mental health web pages. For the intervention group, all accounts will be activated after the research team has verified their identity. Any accounts created after the launch date will be activated as soon as feasible. All participants will then have access to their treatment allocation for 3 months and can choose to engage as frequently as they wish. The control group will receive fortnightly emails resharing the links to the NHS mental health web pages throughout the intervention period.

At the midintervention time point (6 weeks; T_5_), participants will be invited to complete a short online questionnaire via individually generated Qualtrics links. Symptoms of depression (via the PHQ-8) and experiences of AEs and serious AEs (SAEs) will be recorded. Participants will have 2 weeks to respond to the midintervention questionnaire. The research team will send up to 2 email reminders and follow up via telephone if required.

At the postintervention time point (12 weeks; T_6_), accounts on the CommonGround platform will be closed, and the control group will no longer receive emails containing links to the NHS mental health web pages. Participants will be invited to complete the final questionnaire via individually generated Qualtrics links. Participants will have 4 weeks to respond to the postintervention questionnaire. The research team will send up to 2 email reminders and follow up via telephone if required with nonresponders. At any time, if there are any problems delivering the Qualtrics links via email (eg, email undelivered or detected as spam), the research team will attempt to call the participant to resolve the issue (eg, set up a new email address or resend from the study mailbox rather than via Qualtrics automation).

In the 6 weeks after the intervention period (T_6_), a sample of participants (approximately 30 from the intervention arm and 10 from the control arm) will be invited to complete a one-to-one qualitative interview via Microsoft Teams. To explore a range of perspectives, those approached for interview will be purposively sampled based on long-term condition, age, gender, and ethnicity.

### Randomization

#### Overview

Participants will be individually randomized (T_3_) using the King’s Clinical Trials Unit web-based randomization service upon completion of their baseline questionnaires (T_2_) by a research assistant. Online peer support requires a sufficiently large community of active users to enable discussions and foster a community. Therefore, we will allocate participants using a 2:1 ratio (100 to the intervention arm and 50 to the control arm). Treatment allocations will be stratified based on baseline depressive symptom severity using minimization with randomly varying block sizes. Baseline depressive symptom severity will be measured using the PHQ-8 total score (low=PHQ-8 score of <7; high=PHQ-8 score of ≥7). Although the research team will be unblinded to treatment allocations, the web-based system will conceal subsequent allocations.

#### Blinding

Due to the nature of the interventions, it is not possible to blind participants. As the baseline and mid- and postintervention assessments will be self-reported by participants via web-based Qualtrics questionnaires, the outcome assessments will not be blinded. All research team members, including the trial statistician and qualitative interviewers, will be unblinded to treatment allocation.

### Ethical Considerations

This feasibility study (and concurrent observational community cohort) has been approved by the South Central – Oxford C Research Ethics Committee (Integrated Research Application System ID328175). This study is sponsored by KCL. Approval for any amendments will be first sought from our sponsor (KCL) before submission and final approval from the South Central – Oxford C Research Ethics Committee. This trial has been registered on ClinicalTrials.gov (NCT06222346).

Informed consent will be collected on 2 occasions (Multimedia Appendix 3). Interested participants will first consent to the eligibility screenings (at T_1_ and T_2,_ separated by the wait period). If found to be eligible at the second screening, participants will then provide informed consent for the feasibility trial. Participants who consent to the trial will be remunerated with vouchers upon completion of the baseline (£10 [US $13.74]) and postintervention (£10 [US $13.74]) assessments. Those who participate in online interviews will be remunerated with a £20 (US $27.48) voucher. Participants can choose to withdraw from the intervention, or the entire trial, at any time. Those who withdraw from their allocated treatment arm (ie, removal of access to CommonGround or ceasing of the receipt of control emails) but wish to continue with data collection will be contacted to complete the mid- and postintervention questionnaires. Participants will be invited to share their reasons for withdrawal.

All participants will have their right to privacy, communicated and protected at all times. A privacy policy will be drafted for CommonGround. No identifiable information will be published in any output from this fRCT. We will outline our procedures to maintain confidentiality via our participant information sheet with participants before seeking their consent to participate. All individuals who complete the eligibility screenings will be randomly assigned a unique ID. All research data will be pseudonymized, password-protected, and held on secure servers. Data from the one-to-one online interviews or focus groups will be audio recorded and stored in password-protected files on KCL secure servers. Personal data in the form of audio files will also be shared with our transcription service, “Way with Words,” who will sign a nondisclosure agreement prior to listening to any recordings.

### Interventions

#### Intervention Arm: CommonGround

The CommonGround platform has been co-designed through collaboration with people with lived experience of long-term conditions, clinicians, academics, and web design experts. The “all diagnoses welcome” approach, driven by the experiences of our PPI group, aims to be inclusive of all people living with long-term conditions. The online platform has a community feed for forum discussions (peer support component) and a resource section, including evidenced-based self-help information (psychoeducational component). Following usability testing and prototype refinement in collaboration with our RAG (pending publication), CommonGround includes the key features and functions outlined in Textbox 1 [38] (refer to Multimedia Appendix 4 for screenshots).

All participants will be required to provide personal details (name, email address, mobile number, and date of birth) upon creating their account to allow the research team to verify their identities. As participants will be anonymous to one another in the community, they will be asked to create an anonymous pseudonym username. Throughout the intervention period, the research team will be available to assist with any technical or accessibility issues. Participants can engage with CommonGround as often as they wish (ie, participation is voluntary).

**Key features and functions of the CommonGround platform.**

- CommonGround is an online platform that is accessible via desktop and mobile devices. The platform has 2-factor authentication via SMS text message and automatic log-out after 10 minutes of inactivity.
- There is a discussion forum where users can create anonymous posts (eg, “How I am feeling”). Users can also react to (eg, “I hear you” or [laughter emoji]), comment on, and save forum posts.
- The platform includes an intuitive navigation bar that features a notification bell and a search tool to help users locate content of interest.
- Users have access to a private My Garden page, where they can store posts from other users and resources they have chosen to save.
- The Resources page gives access to evidence-based psychoeducational material on the mind-body link alongside self-help information and signposting to reputable resources and further support. The topics were selected for their relevance to people living with *any* long-term condition, such as advice on managing sleep, physical activity, and managing symptoms.
- Users have the ability to follow and mute other users, view other users’ profiles, and read their biography and view their recent activity.
- The platform includes an About Us page where users can read more about the research behind CommonGround, the platform itself, how to contact the research team, and technical troubleshooting (via “how-to” guides).
- There is a Crisis and Further Support page and web page banner where users can find organizations able to provide further support, particularly for crisis situations. This signposting is necessary because one-to-one and crisis support will not be offered on CommonGround.
- Dedicated administrative accounts will have access to an administrative panel and additional moderation functions that will allow our trained moderators to review, edit, and remove content from the community forum. The moderation team will be separate to the research team and solely dedicated to the safety of the platform. The moderators will mediate discussions between users and operate a “three-strike” policy whereby users repeatedly failing to adhere to the community principles (terms of use agreed to upon account creation) will be removed from the platform. Users can also flag posts to the attention of moderators. The moderation, safeguarding, and privacy policies that govern the operation of CommonGround will be clearly displayed on the platform. All policies have been coproduced with our coapplicant and based on guidance from King’s College London research and development and input from established organizations, including the online harms team from the Samaritans [38]. The community principles and moderation and safeguarding policies are available upon request from the corresponding author.
- There will be an engagement team (members of the research team) who will implement the engagement policy that has also been codeveloped with our patient and public involvement group and coapplicant. This policy includes weekly email circulars providing community updates and reminders to those who have not logged in recently. The engagement team will also have administrative accounts to encourage engagement via posts summarizing *trending* topics in the community feed and sharing relevant topics for people living with long-term conditions, which can be pinned to the top of the community feed. The engagement policy is available upon request from the corresponding author.

#### Control Group

From the launch date (T_4_), participants in the control arm will be sent fortnightly emails directing them toward the NHS mental health web pages, a series of web-based resources provided by the NHS [39]. These web pages are not specifically targeted at people living with long-term conditions. The web pages include information sheets and mental health support, including topics such as managing different life situations and events. In addition, there is a self-help section that includes guides, tools, and activities to support mental health. Participants can engage with the NHS content as often as they wish (ie, participation is voluntary).

#### Criteria for Discontinuing or Modifying Allocated Interventions and Posttrial Care

The intervention is intended to complement the usual care participants receive for their long-term conditions. Participants can withdraw from the intervention (complete follow-up assessments) or the entire trial at any time. There are no criteria for discontinuing or modifying allocated interventions. However, for participants in the intervention group, platform access will be removed in the event of severe violations of the community principles and in accordance with the moderation policy. As this feasibility trial is not powered to assess the potential benefits or safety of the platform, there are no planned provisions for ancillary or posttrial care, and all CommonGround accounts will be closed after 3 months (at T_6_). Upon study completion, all participants will be provided with PDFs containing links to the resources from the CommonGround resources page and further support and crisis information.

### Measures and Outcomes

#### Overview

The measures and assessment time points for the CommonGround fRCT are illustrated in Table 1.

**Table 1:** The schedule of enrollment, interventions, and assessments for the CommonGround feasibility randomized controlled trial.

		Study phase							
		Screening (T_1_)	Enrollment (T_2_)	Allocation (T_3_)	After allocation				Closeout (T_6_)
					T_4_	T_5_	T_6_		
**Enrollment**									
	Eligibility screening	✓	✓						
	Informed consent	✓	✓						
	Randomization			✓					
**Interventions**									
	Intervention—CommonGround peer support platform				✓	✓	✓		
	Control—NHS^a^ mental health web pages				✓	✓	✓		
**Assessments**									
	PHQ-8^b^	✓	✓			✓		✓	
	WEMWBS^c^		✓					✓	
	GAD-7^d^		✓					✓	
	SF-36^e^		✓					✓	
	SCS-R^f^		✓					✓	
	CDSES^g^		✓					✓	
	EQ-5D-5L		✓					✓	
	CSRI^h^		✓					✓	
	PAM-13^i^		✓						
	SoVC^j^							✓^k^	
	3-item entitativity questionnaire							✓^k^	
	MAUQ^l^							✓^k^	
	AEs^m^ and SAEs^n^					✓		✓	

^a^NHS: National Health Service.

^b^PHQ-8: Patient Health Questionnaire–8.

^c^WEMWBS: Warwick-Edinburgh Mental Well-Being Scale.

^d^GAD-7: Generalized Anxiety Disorder scale.

^e^SF-36: 36-Item Short Form Health Survey.

^f^SCS-R: Social Connectedness Scale–Revised.

^g^CDSES: Chronic Disease Self-Efficacy Scales.

^h^CSRI: Client Service Receipt Inventory.

^i^PAM-13: Patient Activation Measure.

^j^SoVC: sense of virtual community questionnaire.

^k^Assessment delivered to the intervention group only.

^l^MAUQ: mHealth App Usability Questionnaire.

^m^AE: adverse event.

^n^SAE: serious adverse event.

#### Participant Characteristics

At baseline, we will use web-based Qualtrics questionnaires to collect age, date of birth, gender, sex assigned at birth, ethnicity, long-term physical health conditions, technology and internet use, history of using peer support, attitude toward peer support, and engagement in mental health treatment. The Patient Activation Measure (PAM-13) will also be administered [40]. The PAM-13 will measure the extent to which our sample are more passive care recipients versus more actively engaged in their health care. The PAM-13 is widely used within the NHS and for those living with long-term conditions [41] and has been validated among older adults with multimorbidity [42] and in mental health care settings [43].

#### Feasibility Outcomes

The primary feasibility outcome is the number of participants recruited to the trial per week and in total via each recruitment route (as self-reported by participants). The other feasibility outcomes are (1) number of participants who consent to the eligibility screenings of those contacted by the research team via the consent to contact route (eg, Bioresources and Lambeth NHS Talking Therapies Service), (2) number of participants found to be eligible at first screening (T_1_) of those who consent to the eligibility screenings, (3) number of participants found to be eligible at T_2_ of the number screened at T_1_ and T_2_, (4) number of participants who provide consent to take part in the feasibility trial of those eligible at T_2_, (5) number of participants withdrawn from the trial of those randomized, and (6) number of participants who respond to the follow-up assessment at 3 months (postintervention time point; T_6_) of those randomized.

Table 2 shows the criteria that have been selected to assess the feasibility of the trial. For each criterion, thresholds for *green* (the future definitive trial is likely feasible), *amber* (it may be feasible if appropriate changes are made), and *red* (it is likely not feasible) are presented.

**Table 2:** Feasibility criteria and associated thresholds to inform progression to a larger, future confirmatory randomized controlled trial.

Feasibility criteria	Green	Amber	Red
The number of participants randomized at T_2_^a^ (target: N=150 participants)	N≥120	N≥80	N≥40
Participants found to be eligible by T_2_ of those who consent to the eligibility screenings at T_1_^b^	≥30%	≥20%	≥10%
Participants responding to the follow-up assessment at 3 mo (postintervention time point) of those randomized (T_3_^c^)	≥80%	≥60%	≥40%

^a^Second eligibility screening, consent to trial, and baseline assessment.

^b^Recruitment, consent to take part in eligibility screenings, and first eligibility screening.

^c^Randomization.

#### Acceptability and Usability Outcomes

In the intervention arm only, the acceptability and usability of CommonGround will be measured via the following:

1. Perceived usability at the postintervention time point (T_6_) using the mHealth App Usability Questionnaire (MAUQ). The MAUQ has demonstrated reliability and validity to measure mobile health app usability [44].
2. Qualitative data on the perceived acceptability and benefits of CommonGround to people living with long-term conditions who are experiencing subthreshold depressive disorder.

Platform adherence and use metrics will also be captured in the intervention arm only to provide insights into engagement. This will include the clicks and interactions of participants when accessing and using the CommonGround platform, specifically the (1) number of times logging into the platform (in total, per day, and per week), overall and by device type (eg, mobile and desktop); (2) time spent logged in (in total, per day, and per week), overall and by device type (eg, mobile and desktop); (3) number of original posts created in the community feed, comments on posts, reactions to posts, and searches; (4) number of user-to-user interactions, including follows and unfollows, muting, viewing other users’ profiles, and posts flagged; (5) number of posts edited or deleted by moderators and time spent moderating; and (6) number of times participants download or open resources and save content to the My Garden private page.

#### Clinical and Health Economic Outcomes

Clinical outcomes will be measured for all participants at baseline (T_2_) or the postintervention time point after the 3-month intervention period (T_6_). All clinical outcomes will be treated as exploratory as this feasibility trial is not powered to evaluate clinical efficacy. These outcomes are (1) incidence of probable major depressive disorder at the postintervention time point only (T_6_), measured using the PHQ-8 [30] (PHQ-8>9=1; PHQ-8≤9=0); (2) symptoms of depression at the midintervention (week 6; T_5_) and postintervention (T_6_) time points, measured using the PHQ-8 total score [30]; (3) well-being at the postintervention time point (T_6_), measured using the Warwick-Edinburgh Mental Well-Being Scale [31]; (4) symptoms of anxiety at the postintervention time point (T_6_), measured using the Generalized Anxiety Disorder scale total score [45]; (5) health-related quality of life at the postintervention time point (T_6_), measured using the 36-Item Short Form Health Survey [46]; (6) social connectedness at the postintervention time point (T_6_), measured using the Social Connectedness Scale–Revised [47]; (7) self-efficacy in managing their health at the postintervention time point (T_6_), measured using the Chronic Disease Self-Efficacy Scales [48]; and (8) sense of virtual community at the postintervention time point (T_6_), measured using the sense of virtual community questionnaire [49] and an adapted 3-item entitativity measure [50,51].

The following measures will be used for the health economic outcomes:

1. The EQ‐5D‐5L, a standardized measure of health status consisting of 5 dimensions [52]. Standard value sets are summarized using a single index value determining the valuation of health-related quality of life [53].
2. The Client Service Receipt Inventory [54]. We will use an adapted version of the Client Service Receipt Inventory that measures services and supports that a participant may have used over the previous 3 months. Subsections include accommodation and living situation, employment history, health care service use, and informal support.

#### Qualitative Outcomes

A nested qualitative study will be conducted for this feasibility trial. Within 6 weeks after the intervention (T_6_), a selection of participants will be invited to schedule a qualitative interview. For those who were in the intervention arm, the interviews will investigate their experiences and views on the peer support platform, examining the acceptability of the intervention and its perceived benefits and limitations in relation to the preconditions for change identified in the theory of change model. For all participants, the acceptability of trial procedures, including views on randomization and the relevance and burden of outcome measures, will also be explored. For those who were in the control arm, their perspectives on the acceptability of the control condition will be explored. Interview topic guides will be coproduced with our PPI group and coapplicant with lived experience. The online interviews will be audio recorded to enable transcriptions to be produced. The interviews will be conducted with approximately 40 individual participants (30 from the intervention arm and 10 from the control arm), which we estimate will afford sufficient information power [55].

The moderation team will also be invited to participate in an online focus group to explore their experiences moderating the CommonGround platform during the intervention period in accordance with the policies. Capturing moderators’ experiences will provide a unique perspective on how users interact with the platform. It will also provide insight into any safeguarding issues or risks and their management to inform future iterations of the trial and the associated moderation policies and procedures.

### AE Management

AEs and SAEs will be captured throughout the 3-month trial period. This includes the following:

1. Via a self-report questionnaire administered to participants in both arms at the midintervention (6 weeks; T_5_) and postintervention (12 weeks; T_6_) time points. This includes self-reporting of experiences of a list of expected AEs that we will coproduce with our PPI group.
2. Through monitoring and recording any other SAEs and AEs that occur throughout the intervention period.
3. As per recommended guidance on assessing the safety of digital health interventions [56] monitoring depressive symptom deterioration (as per the PHQ-8 score). The progression from mild depressive symptoms at baseline (score of 5-9) to either moderate (score of 10-14) or moderately severe (score of 15-19) depressive symptoms will also be classified as an expected AE. Progression to severe depressive symptoms (score of ≥20) will be classified as an expected SAE.

The AE protocol for this feasibility trial, including definitions, categorizations for expectedness and relatedness, and reporting procedures will be published elsewhere. Adequately capturing AEs and SAEs among a sample living with long-term conditions and subthreshold depressive disorder is important given that certain events (eg, hospitalization) are more likely to occur in our sample than in a healthy sample.

### Nonadherence

In the intervention arm, nonadherence to the CommonGround intervention will be assessed based on those who (1) are randomized to the intervention arm but do not create an account, (2) create an account but do not log in, or (3) log in less than once a month. In the control arm, nonadherence will be assessed through self-report, where participants will be asked whether they accessed the NHS mental health web pages. In both arms, reasons for nonadherence will be explored in the qualitative interviews. Nonadherence to completion of the baseline, midintervention, and 3-month follow-up questionnaires will be considered if participants do not respond to or complete the questionnaires.

### Data Management and Trial Monitoring

The procedures to maintain participant confidentiality will be outlined in the participant information sheet before consent. All individuals who complete the eligibility screenings will be randomly assigned a unique ID. Those randomized to CommonGround will be anonymous to other participants under their chosen pseudonym usernames. Such anonymity will be enforced by the moderation team, who will remove personally identifiable information from any forum posts or comments. Participants will be notified that anonymity may be broken by the research team under the circumstances required by the moderation and safeguarding policies (eg, disclosure of intent to commit terrorism offense or safeguarding concerns). Any use metrics or CommonGround account data shared between the software developer (Bitjam; data processor) and KCL (data controllers) external to the CommonGround platform will be pseudoanonymized (using the unique study IDs). Interview audio recordings will be shared securely with a transcription service and deleted thereafter. Platform data, data extracted from Qualtrics, and interview audio recordings or transcripts will be password protected and stored on secure servers.

A trial steering committee (TSC) will be formed, including an independent chair and independent members, the principal investigator, and the trial manager and statistician. The TSC will meet 3 times during the trial. For data monitoring purposes, we will also have an executive committee (with a single external member) who will review safety information (AEs and SAEs split by arm) and make recommendations to the blinded TSC chair, including recommendations regarding the continuation of the trial at the midintervention time point (T_5_).

### Statistical Analysis

We will follow the CONSORT (Consolidated Standards of Reporting Trials) 2010 statement extension for randomized feasibility and pilot trials [57] For this protocol, we have followed the SPIRIT (Standard Protocol Items: Recommendations for Interventional Trials) checklist (Multimedia Appendix 5). We will describe feasibility outcomes, use metrics, and clinical outcomes using appropriate summary statistics (eg, mean and SD, median and IQR for normally distributed continuous outcomes, and counts and proportions for categorical outcomes). As the clinical outcomes have been selected for their relevance for informing potential effect sizes for a future confirmatory trial, regression models will be used to estimate the effect of treatment allocation on outcomes measured at the postintervention time point (T_6_). The analysis of clinical outcomes will follow a modified intention-to-treat principle. All eligible, randomized participants with a recorded outcome (ie, the outcome is not missing) will be included in the analysis and analyzed according to the treatment to which they were randomized. For binary outcomes (incidence of probable major depressive disorder at the postintervention time point), we will conduct a binary logistic regression where the model includes terms for treatment allocation (peer support platform=1; control arm=0), the baseline severity minimization factor (high=1; low=0), and the baseline value of the outcome. If the binary outcome of major depressive disorder is common (eg, >20%), we will consider robust Poisson models instead, following the work by Zou [58]. For linear outcomes (eg, symptoms of depression at the postintervention time point), we will use linear regression with the same covariates. Exploratory treatment effects will be estimated based on the coefficients for treatment allocation; 95% CIs will be estimated using bootstrapping. Given the sample size and purpose of this feasibility trial, all estimates of treatment efficacy will be treated as underpowered and not used as the basis for inferential statements. To inform sample size estimation for a future confirmatory trial, we will calculate the SD of the continuous clinical outcomes.

### Qualitative Analysis

For the participant interviews, framework analysis [59] will be used to facilitate analysis within and between individual cases and groups of participants. Members of the research team will independently code the first 5 interview transcripts and discuss alternative viewpoints before agreeing to a provisional analytical framework to be applied to subsequent transcripts. The thematic framework will draw on a priori issues identified in the theory of change model as key factors related to acceptability and usability of the CommonGround platform and trial procedures while being responsive to any emergent themes. The wider research team will meet regularly to examine coding and preliminary interpretations to ensure that all themes are firmly rooted in the data. We will discuss theme names, descriptions, and illustrative quotes with the RAG to further enhance the trustworthiness of the findings. Once applied to individual transcripts, data will be charted to map the dataset as a whole, allowing the RAG and the wider research team to engage with and offer interpretations of the data. Quantitative measures related to the community (eg, sense of virtual community and entitativity) will be interpreted alongside participants’ qualitative accounts of whether they felt connected with their peers and experienced a sense of community. In addition, use metrics (eg, number of log-ins, comments, and other interactions) will be examined in relation to participants’ descriptions of the features that they engaged with the most and found most useful or enjoyable. For usability, scores on the MAUQ will be considered in conjunction with participants’ interview accounts of their experiences navigating the platform, including any barriers to or facilitators of use.

Integration of these data will be carried out collaboratively by the qualitative and quantitative researchers, with discussion to explore patterns, contradictions, and complementary findings across data types. This triangulation will support a more nuanced understanding of participant engagement and user experience and will help identify priorities for refinement of the intervention and trial procedures.

### Dissemination

We will hold workshops with our RAG to explore ways of sharing our results with patients and the public. We will also publish our results in peer-reviewed journals and present this work at key meetings or conferences.

## Results

Recruitment for this feasibility trial began on February 12, 2024. We randomized 125 participants. Quantitative data collection was completed on October 23, 2024, and qualitative data collection was completed on December 3, 2024.

## Discussion

### Expected Findings

This trial will explore the acceptability and feasibility of CommonGround, a coproduced online peer support platform with embedded psychoeducational resources. The platform is purposefully targeted at people living with long-term physical health conditions and subthreshold depression who are at risk of developing major depressive disorder. Adopting a proactive, preventative approach to depression that intervenes earlier than the mainstream reactive approach currently used in the NHS could improve lives and reduce the burden of comorbid mental illness on patients, families, the health care system, and society.

As online peer support is accessible, inexpensive, and scalable, CommonGround might offer a way of bridging the gap in psychosocial care for people living with long-term conditions. Our intervention has been carefully coproduced through iterative prototype developments with our PPI and RAG groups, our coapplicant with lived experience, software developers, and clinicians and academics. This coproduction maximizes the potential for our intervention to address the unmet psychosocial needs of people living with long-term conditions explored in our previous work [29]. The “all diagnoses welcome” ethos of the CommonGround peer support platform contrasts with the traditional condition-specific siloed approach in the provision of peer support. While our prior work has highlighted how people with long-term conditions share common experiences that transcend diagnoses, it will be important to consider our findings in the context of a diverse patient sample and explore the acceptability of this ethos among a larger sample in the nested qualitative study.

The data derived from this feasibility trial will be essential to informing the completion of a larger multisite trial exploring the potential clinical efficacy and cost-effectiveness of CommonGround. We estimate that such a confirmatory trial would be completed within 42 months of the completion of this trial. If future confirmatory trials indicate the efficacy and safety of CommonGround, the intervention could be deployed in NHS services and benefit patients within 5 years. Such implementation would represent an efficient use of NHS health care resources, particularly as the “all diagnoses” inclusive approach will penetrate the NHS far more readily than a system that is distinct for each long-term condition. The platform’s online, remote nature will likely ensure that rapid scale-up and wider implementation are easily achieved.

### Limitations

Adoption of the intervention in routine care would require careful planning of referral strategies and a scaled-up moderation strategy, including recruitment, training, and support of a larger team of peer or clinical moderators. Large-scale adoption would also require long-term investment in hosting and maintaining platform software to support a larger community and volume of activity. It is possible that the software developer agency will cease to operate or that technical issues will occur. Therefore, we are ensuring that the platform is built using open-source software that can be maintained and adapted by any software developer with appropriate skills.

The online nature of the intervention and trial allows participants to become involved in peer support regardless of geographical location within the United Kingdom and proximity to physical services or support groups, offering a means of accessing evidence-based, reviewed self-management resources remotely. The online nature also allows participants to use CommonGround as they wish regarding the self-management of their condition and potentially fluctuating symptoms. However, this requires participants to have consistent access to both the devices and reliable internet connection required to successfully participate in the CommonGround community and research trial. While >96% of households in Great Britain have internet access [60], patients who live in households that do not have access to the internet might find this to be a barrier to enroll in the study or engage with the online intervention. The inclusion criteria of having access to the internet does not specify that this internet access must be in the home, and therefore, participants can engage from other spaces where they might be able to access the internet for free (eg, public libraries). Finding the time and space to engage with the research might be more difficult for those who do not have internet access within the home, and therefore, although it is not possible to explore methods of overcoming such barriers within the context of this feasibility trial, this could be the focus of future work to enhance access equity.

Anticipated challenges in running the intervention relate to forgotten passwords or issues logging in and technical bugs or glitches. To minimize the chance of these factors influencing engagement, there will be various “how-to” troubleshooting guides published on the platform, and both KCL and the software developers will have standardized procedures to diagnose and resolve technical issues in a timely manner. A final limitation of this feasibility trial is that, due to the nature of the intervention, it is impossible to blind participants or the study team. Due to the 2:1 design, it is not possible to blind the study statistician.

### Conclusions

This trial will provide important feasibility and acceptability data on a coproduced “all diagnoses” welcome online peer support platform with embedded psychoeducation. It will inform the design of a future confirmatory trial, the essential next step in the potential for CommonGround to support the psychosocial well-being and self-management of people living with a variety of long-term physical health conditions who are at risk of developing major depressive disorder.

## Appendix

Multimedia Appendix 1 List of long-term physical health conditions presented to interested individuals when completing the eligibility screenings.

Multimedia Appendix 2 Protocol for the community engagement cohort.

Multimedia Appendix 3 Consent forms for the eligibility screenings and trial enrollment for the CommonGround feasibility randomized controlled trial.

Multimedia Appendix 4 Screenshots of the CommonGround online peer support platform.

Multimedia Appendix 5 SPIRIT checklist for the CommonGround feasibility randomized controlled trial.

## Abbreviations

AE: adverse event
CONSORT: Consolidated Standards of Reporting Trials
fRCT: feasibility randomized controlled trial
IMPARTS: Integrating Mental and Physical Healthcare: Research Training and Services
KCL: King’s College London
MAUQ: mHealth App Usability Questionnaire
NHS: National Health Service
PAM-13: Patient Activation Measure
PHQ-8: Patient Health Questionnaire–8
PPI: patient and public involvement
PTSD: posttraumatic stress disorder
RAG: research advisory group
RCT: randomized controlled trial
SAE: serious adverse event
SPIRIT: Standard Protocol Items: Recommendations for Interventional Trials
TSC: trial steering committee

## Data Availability

The datasets generated or analyzed during this study are available from the corresponding author on reasonable request.
