# Supplementary material for: Online Peer Support for Long-Term Conditions: Protocol for a Feasibility Randomized Controlled Trial
Source: JMIR Res Protoc. 2025 Jul 23;14:e71513. doi: 10.2196/71513 (PMC12329384; doi:10.2196/71513)
Supplement: Multimedia Appendix 1 [file resprot_v14i1e71513_app1.docx]

# Multimedia Appendix 1.

The following list of LTCs will be presented to people completing the eligibility screenings as examples of common LTCs. The option of ‘Other’ will also be provided. Respondents will be provided with contact details of the research team if they have any queries or wish to discuss the eligibility criteria in further detail.

- Asthma
- Atrial fibrillation
- Cancer
- Cerebral palsy
- Cerebrovascular disease (includes stoke and transient ischaemic attack)
- Chronic kidney disease stages 3-5
- Chronic primary headache
- Chronic primary musculoskeletal pain
- Chronic primary visceral pain
- Chronic obstructive pulmonary disease
- Complex Regional Pain Syndrome
- Coronary heart disease (includes angina, heart attack)
- Crohn’s disease
- Cystic fibrosis
- Diabetes (Type I)
- Diabetes (Type II)
- Endocrine disorders (thyrotoxicosis, hypothyroidism, hypogonadism, Cushing syndrome, Addison’s disease)
- Endometriosis
- Epilepsy
- Fibromyalgia
- Heart failure
- Human immunodeficiency virus; acquired immune deficiency syndrome
- Hypertension
- Inflammatory Bowel Disease
- Irritable bowel syndrome
- Liver disease
- Lupus
- Migraine
- Multiple sclerosis
- Orofacial pain
- Osteoarthritis
- Osteoporosis
- Parkinson’s disease
- Peripheral Arterial Disease; Peripheral Vascular Disease
- Psoriasis
- Polycystic ovary disease
- Rheumatoid arthritis
- Sickle cell anaemia
- Viral hepatitis (B&C)
- Other (please specify)
