# Supplementary material for: Online Peer Support for Long-Term Conditions: Protocol for a Feasibility Randomized Controlled Trial
Source: JMIR Res Protoc. 2025 Jul 23;14:e71513. doi: 10.2196/71513 (PMC12329384; doi:10.2196/71513)
Supplement: Multimedia Appendix 2 [file resprot_v14i1e71513_app2.docx]

# Multimedia Appendix 2. Protocol for the community engagement cohort

# Title:

Online peer support in long-term conditions: A feasibility randomised controlled trial (Community Engagement)

Abbreviations.

| CSRI | Client Services Receipt Inventory |
| --- | --- |
| EQ‐5D‐5L | EuroQol EQ-5D-5L descriptive system |
| fRCT | Feasibility randomised controlled trial |
| GAD-7 | Generalized Anxiety Disorder assessment |
| GPs | General Practitioners |
| IAPT | Improving Access to Psychological Therapies |
| ID | Identification |
| KCL | Kings College London |
| LTCs | Long-term conditions |
| NHS | National Health Service |
| PAM-13 | Patient Activation Measure |
| PHQ-8 | Patient Health Questionnaire-8 |
| PTSD | Post Traumatic Stress Disorder |
| SF-36 | Medical Outcomes Study Questionnaire Short Form-36 |
| SMS | Short Messaging Service |

# Introduction.

The current community engagement study runs parallel to a feasibility randomised controlled trial (fRCT) run by King’s College London (Title: Online peer support in long-term conditions: A feasibility randomised controlled trial; Trial Registration: ClinicalTrials.gov: NCT06222346). In brief, the fRCT aims to investigate the feasibility of conducting a larger confirmatory trial that assesses the clinical efficacy and cost effectiveness of a new peer support platform with embedded psychoeducational resources that has been co-produced between academics, software developers, and groups of people living with long-term conditions.

The fRCT requires an individual to score between 5 and 9 on the PHQ-8 to participate. However, given how mental health and depressive symptoms may vary over time, we anticipate having potential participants who score 0-4 or 10-14 on the PHQ-8, who do not have a diagnosis of a severe mental health condition, but are experiencing some depressive symptoms. Such group may also benefit from online peer support catered for people with psychological distress in the context of chronic physical illness, including our CommonGround intervention described in the fRCT protocol. For new online communities to develop the conversations, discussions, and user-to-user interactions that underlie peer support, the community needs to be sufficiently large. Given observations that online communities often consist of a smaller subgroup of active users generating original content and a larger subgroup of users who are observers of the content shared, having a larger cohort onboarded onto the new peer support platform increases the probability of a higher volume of content shared in the community feed. Therefore, to facilitate the emergence of peer support in the fRCT, a second group of people living with long-term conditions and scoring either 0-4 or 10-14 on the PHQ-8 will be offered the opportunity to participate in a Community Engagement Study and be onboarded onto the platform alongside the participants randomised to the intervention arm of the fRCT.

The aims and objectives of this Community Engagement Study align with those of the fRCT: to examine the feasibility and acceptability of a co-produced peer support platform for people living with LTCs and depressive symptoms. The specific objectives are outlined in the fRCT protocol.

# Methods: Participants, interventions, and outcomes.

## Participants.

All participants will be recruited in the United Kingdom by King’s College London after expressing their interest and completing eligibility assessments to participate in the fRCT assessing the feasibility of a new online peer support platform to manage symptoms of depression in people living with long-term conditions. The routes of recruitment are described in full in the fRCT protocol. In brief, recruitment routes include outpatient clinics at King’s College Hospital NHS Foundation Trust and Guy’s and St Thomas’s NHS Foundation Trust; other consent to contact patient databases (e.g., BioResource database; IAPT Lambeth talking therapies); via charities or patient organisations, including their social media channels; and poster advertisements in appropriate locations (e.g., GP clinics), and snowballing via word-of-mouth.

Individuals who have completed the two eligibility screenings (#1 at the first point of contact; #2 after the wait period whilst the desired sample size is recruited to allow all participants to be onboarded simultaneously) but are not eligible to participate in the fRCT (due to their PHQ8 scores *only*) will be offered the opportunity to participate in this Community Engagement Study. Consent to participate in the Community Engagement Study is separate to consent for the previous eligibly screenings, and a separate information sheet will be provided at the time of offer to participate. The flow of participants from recruitment through to study completion is illustrated in Figure 1.

**Figure 1.** Flow diagram illustrating the flow of participants from recruitment through to data collection and analysis, highlighting the point at which participation in the Community Engagement study is offered and how the participants are combined with participants randomised to the intervention arm of the fRCT


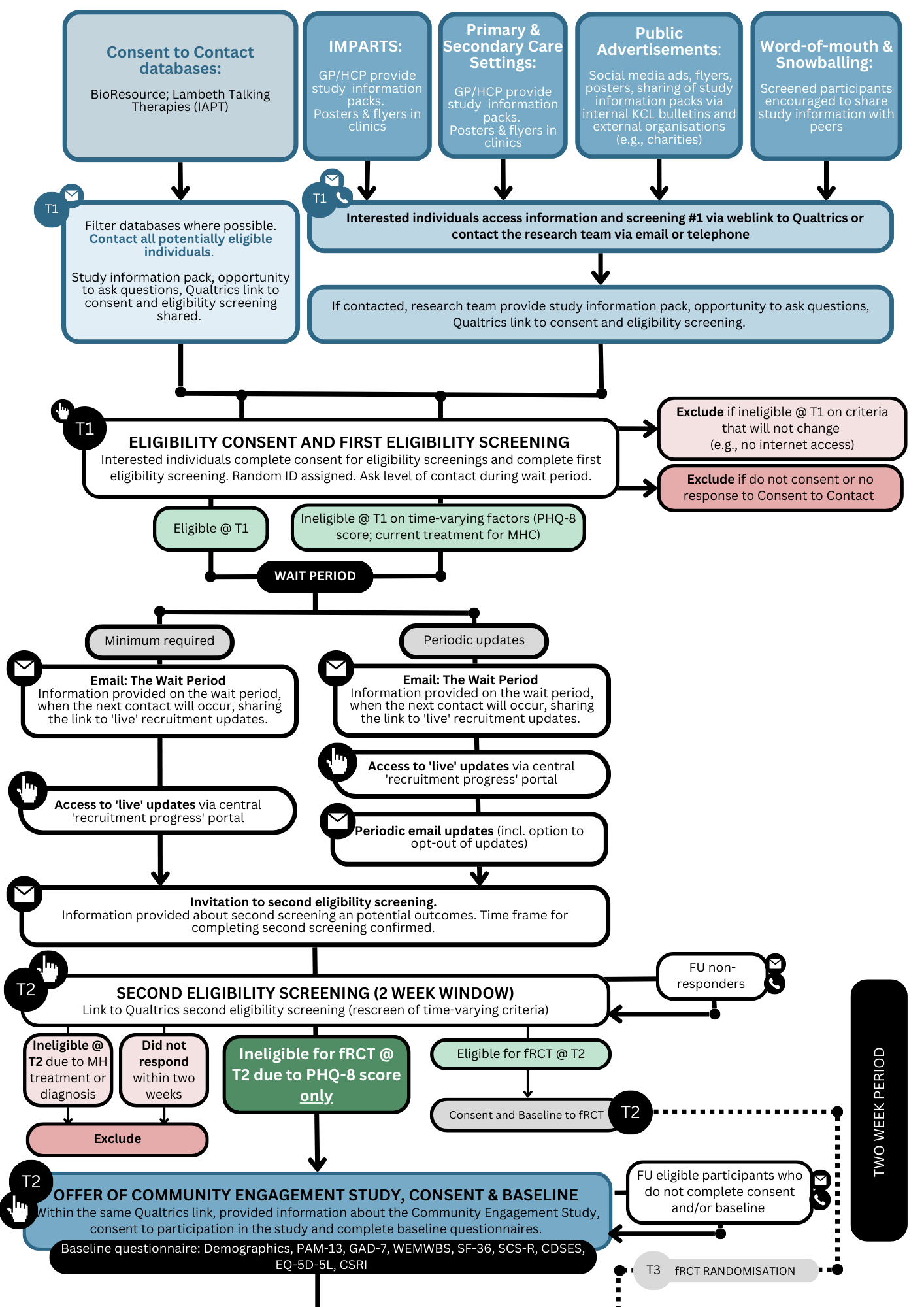


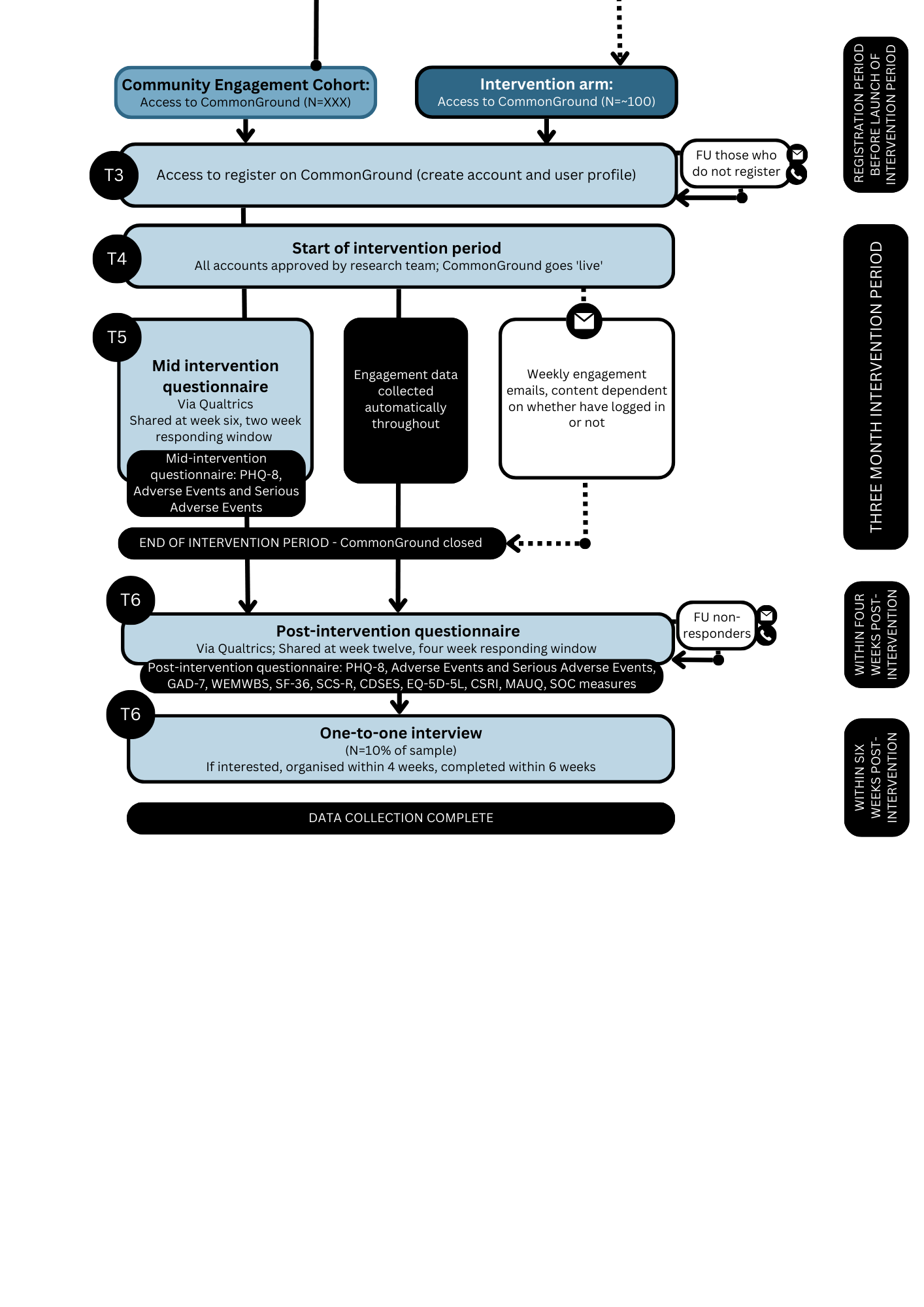


*FU: Follow up; IAPT: Improving Access to Psychological Therapies; IMPARTS: Integrating Mental & Physical healthcare: Research, Training & Services; KCL: King’s College London; MHC: Mental health condition; PHQ-8: Patient Health Questionnaire-8*

## Eligibility criteria.

| **Inclusion criteria** |
| --- |
| - Living with long-term physical health condition(s). A long-term condition is a diagnosed physical medical condition with a duration of ≥6 months that demonstrates recurrence or deterioration and is typically associated with a poor prognosis (O’Halloran, 2014). There are no restrictions on the type of long-term condition. Examples include cerebral palsy, diabetes, and multiple sclerosis. - Aged ≥18 years old. - Access to the internet (via phone, desktop, and/or laptop; within or outside your home, including via mobile data). - Sufficient English to engage with the platform. - Able to give informed consent. - PHQ-8 scores of 0-4 (none to minimal depressive symptoms) or 10-14 (moderate depressive symptoms). |
| **Exclusion criteria** |
| - Have ever received a clinical diagnosis of severe mental illness of bipolar, psychosis, post-traumatic stress disorder, and/or schizophrenia, and/or a diagnosis of dementia. |

This cohort and the participants of the fRCT differ only on their PHQ-8 scores at baseline, whereby the fRCT participants have sub-threshold depressive symptoms (PHQ-8 score of 5-9). Participants from both studies will form a single online community and provide peer-to-peer support. Participants will be unable to distinguish whether people they interact with are part of the Community Engagement study or the fRCT.

## Timeline and recruitment.

The timeline for participants of the Community Engagement Study is illustrated in Figure 2. Participants who are not eligible for the fRCT after completing the second eligibility screening will be provided information about the Community Engagement Study and invited to participate. Participants can then consent to participate and complete the baseline questionnaires via the same Qualtrics link. During a two-week window before the start of the intervention period, participants will be invited via email to register and create their CommonGround profile. When the intervention period begins, the research team will approve all registered accounts, granting access to the peer support platform. All accounts require approval by the research team to verify they belong to a genuine research participant, ensure usernames are anonymous, and link their unique trial ID. Participants can then use CommonGround, the online peer support platform, as they wish for three months (i.e., there are no requirements for the number of logins, etc). After three months, participants will not be permitted to use the site, and all CommonGround accounts will be closed. Participants will complete a short online questionnaire at 6-weeks (mid-intervention; two-week response window) and a series of questionnaires at post-intervention (four-week response window) via Qualtrics. A sample of participants will also be invited to complete a one-to-one virtual interview exploring their views of the intervention itself and the study procedures (organised within six weeks post-intervention).

**Figure 2.** Timeline for participants, timepoints of assessment and measures assessed.


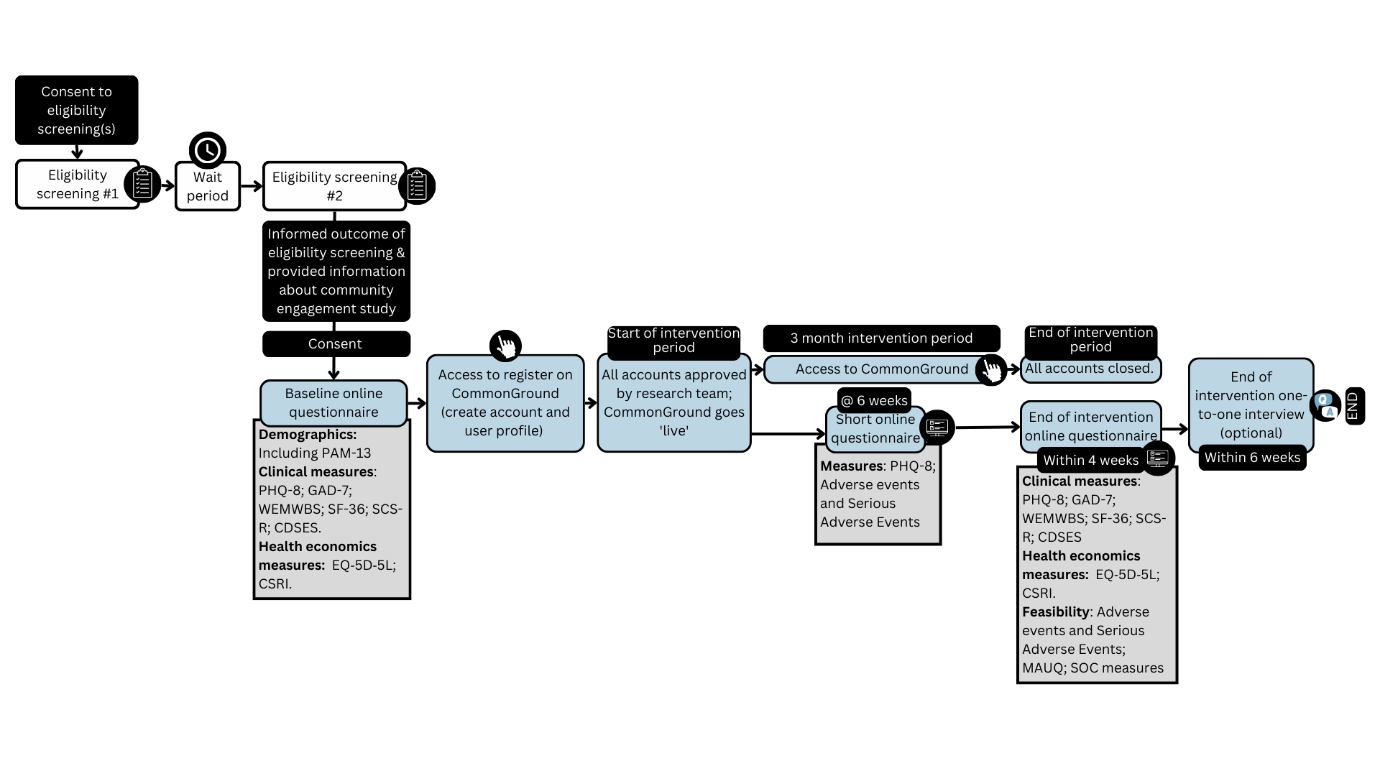


*AEs: Adverse events; CDSES: Chronic Disease Self-Efficacy Scales; CSRI: Client Services Receipt Inventory; EQ-5D-5L: EuroQol EQ-5D-5L descriptive system; GAD-7: Generalised Anxiety Disorder assessment; MAUQ: mHealth App Usability Questionnaire; NHS: National Health Service; PAM-13: Patient Activation Measure; PHQ-8: Patient Health Questionnaire-8; SAEs: Serious adverse events; SCS-R: Social Connectedness Scale-Revised; SF-36: 36-item Short Form Survey; SoVC: Sense of virtual community; WEMWBS: Warwick Edinburgh Mental Wellbeing Scale*

## Intervention.

CommonGround has been co-designed by the research team, software developers, and people living with long-term condition(s). During development, extensive usability testing (Think Aloud exercises; one-to-one interview) informed further development and refinement of the online platform. The intervention includes a ‘Community Feed’ discussion forum and ‘Resources’ page that includes psychoeducational evidence-based information and self-help advice. The platform has been carefully created to be inclusive of anyone living with *any* long-term condition (i.e., regardless of their diagnosis). The web-based platform can be accessed via desktop and mobile devices and has two-factor SMS authentication and automatic logout after 10 minutes. The key features of the platform include:

- A discussion forum where users can create anonymous posts (e.g., ‘How I am feeling’) and react to (e.g., ‘I hear you’, ‘laughter emoji’) and comment on posts from other users.
- An intuitive navigation bar and search tool to locate content and posts of interest.
- A private “My Garden” page that stores posts from other users and psychoeducation content that the user has decided to ‘save’.
- A “resources” page giving access to evidence-based psychoeducation material on the mind-body link (e.g., advice on managing sleep, physical activity, managing symptoms). The “resources” page also contains self-help strategies and signposts to other reputable resources and sources of support.
- The ability to ‘follow’ and ‘mute’ other users, view their profiles, and to read their biography and recent activity.
- An ‘About Us’ page where users can read more about the research, the platform itself, and how to contact the research team.
- A ‘Crisis and Further Support’ page, where users can find further organisations and sources of support, particularly for crisis situations. The peer support platform does not offer one-to-one or crisis support.
- An Admin panel and additional moderation functions that allow our moderator(s) to review, edit, and remove content. The unique administration account(s) will mediate discussions between users and operate a ‘three strike’ policy whereby users repeatedly failing to adhere to community guidelines will be removed from the platform. Users can also ‘flag’ posts to the attention of moderator(s).

During the three-month intervention period, the platform will operate in accordance with a Moderation Policy, Safeguarding Policy, and Engagement Policy, which have all been co-produced with our co-applicant with lived experience based on guidance from KCL R&D. Participants will agree to the Community Principles (‘terms of use’) when creating their CommonGround account, which the Moderation Policy operates in accordance with. Both the Community Principles and Moderation Policy have been reviewed by the Samaritans Online Harms Team. Participants will be anonymous to all other participants on the CommonGround platform; however, the research team will be able to link personal details to anonymous usernames under the circumstances outlined in the Moderation and Safeguarding Policies. The Engagement Policy aims to promote participants to engage with the peer support platform and includes emailing participants on a weekly basis, engaging with those who have been online, and encouraging those who have not logged in within the past week. The engagement team will also encourage engagement via posts written summarising ‘trending’ topics in the ‘Community Feed’ and using prompts and topics considered relevant for people living with long-term conditions.

The continuation of usual care is encouraged throughout the intervention period, as the intervention is intended to complement the usual care an individual is receiving for their long-term condition(s). Participants can request to withdraw from the intervention at any time, resulting in the closure of their CommonGround account. Participants can also choose to not log in to the platform. All participants will be contacted to complete mid- and post-intervention questionnaires unless a participant has explicitly requested to withdraw from the data collection processes.

## Outcomes.

### Participant characteristics.

The characteristics collected at baseline will include age/date of birth, gender, sex at birth, ethnicity, marital status, education, diagnosis, technology and internet usage, previous peer support usage and attitude, and mental health history/treatment. Participants will also complete the Patient Activation Measure (PAM-13) (Hibbard, Mahoney et al. 2005), a measure of the extent to which our sample are more passive recipients of care versus more actively engaged in their healthcare. The PAM-13 is widely used within the NHS and for those living with chronic diseases (Tusa, Kautiainen et al. 2020). The PAM-13 has been validated among multimorbid older adults (Skolasky, Green et al. 2011) and those with mental health conditions.

### Feasibility outcomes.

1. The number of participants consenting to participate in the Community Engagement Study versus those offered the opportunity to participate in the Community Engagement Study at T_2_
2. Number of participants withdrawn from the study of those consenting to participation.
3. Number of participants responding to follow-up questionnaires at three months (post-intervention), of those who consented.
4. Number of participants consenting to the Community Engagement Study from each of the recruitment routes used.

### Platform adherence and usage metrics.

We will collect data measuring engagement with the peer support platform. This includes clicks and interactions participants make when accessing and using the platform, specifically:

[1] Number of times logging into the platform (total and per day/week), overall and by device type (e.g., mobile, desktop).

[2] Time spent logged in (total and per day/week), overall and by device type (e.g., mobile, desktop).

[3] Number of (i) original posts in the community feed; (ii) replies to posts; (iii) reactions to posts; and (iv) number of searches.

[4] Number of user interactions, including (i) follows/unfollows; (ii) ‘muting’; (iii) viewing other members’ profiles; (iv) number of posts flagged.

[5] Number of posts edited or deleted by moderators and time spent moderating.

[6] Number of times participants (i) download or open resources; and (ii) save content to the ‘My Garden’ private page.

### Clinical outcomes.

All clinical outcome data will be self-reported via online questionnaires through Qualtrics, besides the one-to-one qualitative interviews. All clinical outcomes will be treated as exploratory and for their relevance to a future confirmatory trial. The exploratory clinical outcomes that will be measured are:

(1) Incidence of probable Major Depressive Disorder (MDD) at 3-month follow-up only, measured by the PHQ-8 (Shin, Lee et al. 2019) (PHQ-8 >9 = 1; PHQ-8 ≤9 = 0).

(2) Symptoms of depression at mid-intervention (Week 6) and 3-month follow-up, measured by PHQ-8 total score.

(3) Symptoms of anxiety at 3-month follow-up, measured by Generalized Anxiety Disorder assessment (GAD-7) (Spitzer, Kroenke et al. 2006) total score.

(4) Well-being at 3-month follow-up, measured by the Warwick-Edinburgh Mental Well-Being Scale (WEMWBS) (Tennant, Hiller et al. 2007).

(5) Health-related quality of life at 3-month follow-up, measured by the Medical Outcomes Study Questionnaire Short Form-36 (SF-36) (Ware and Sherbourne 1992).

(6) Social connectedness at 3-month follow-up, measured by the Social Connectedness Scale–Revised (SCS-R) (Lee, Draper et al. 2001).

(7) Self-efficacy at 3-month follow-up, measured by the Chronic Disease Self-Efficacy Scales (CDSES) (Lorig, Stewart et al. 1996).

(8) Sense of virtual community at 3-month follow-up, measured by the Sense of Virtual Community questionnaire (SoVC) (Blanchard 2007) and an adapted 3-item Entitativity measure (Blanchard, Caudill et al. 2020, Smith, Alam et al. 2022).

### Health economic outcomes.

(9) The EQ‐5D‐5L, a standardised measure of health status, consisting of five dimensions (Herdman, Gudex et al. 2011). Standard value sets are summarised using a single index value determining the valuation of health-related quality of life (Devlin, Shah et al. 2018).

(10) Client Services Receipt Inventory (CSRI). We will use and adapted version of the Client Services Receipt Inventory (Beecham 1992) that measures services and supports that a participant may use over the past 3-month period. Subsections include background demographic information, accommodation and living situation, employment history, health-care service use, and informal supports.

### Acceptability and usability outcomes.

(8) Perceived usability at 3-month follow-up, measured using mHealth App Usability Questionnaire (MAUQ). The MAUQ has demonstrated reliability and validity to measure mHealth App usability (Zhou, Bao et al. 2019).

### Qualitative outcomes.

A sample of participants will be invited to participate in a one-to-one virtual interview after the three-month intervention period (to be organised within 6-weeks post-intervention). We intend to conduct interviews with a small subset of the sample (specific subset to be confirmed once the community engagement cohort is recruited). The interview will explore participants’ experiences and views of the peer support platform, including acceptability of the peer support platform and any perceived benefits and limitations. Those who have moderated the peer platform during the 12-week intervention will also be invited to take part in a focus group. Capturing moderators’ experiences will provide a unique perspective on how users interact with the platform and any safeguarding issues around risks and their management to inform future iterations of the trial. Moderators will be asked to provide demographic information also. Inclusion criteria include i) Experience moderating our “CommonGround” platform ii) 18 years and over iii) Ability to communicate in and understand English. Those who are moderating the platform will be directly contacted via email by the research team inviting them to take part. We aim to recruit 2-5 people to take part in this focus group.

## Data management, statistical methods, oversight and monitoring, adverse event reporting and harms and dissemination plans.

The above sections are outlined in detail in the protocol for the fRCT.

**References**

Beecham, J., Knapp M. (1992). "Costing psychiatric interventions." Measuring Mental Health Needs.

Blanchard, A. L. (2007). "Developing a sense of virtual community measure." Cyberpsychol Behav **10**(6): 827-830.

Blanchard, A. L., L. E. Caudill and L. S. Walker (2020). "Developing an entitativity measure and distinguishing it from antecedents and outcomes within online and face-to-face groups." Group Processes & Intergroup Relations **23**(1): 91-108.

Devlin, N. J., K. K. Shah, Y. Feng, B. Mulhern and B. van Hout (2018). "Valuing health-related quality of life: An EQ-5D-5L value set for England." Health Econ **27**(1): 7-22.

Herdman, M., C. Gudex, A. Lloyd, M. Janssen, P. Kind, D. Parkin, G. Bonsel and X. Badia (2011). "Development and preliminary testing of the new five-level version of EQ-5D (EQ-5D-5L)." Qual Life Res **20**(10): 1727-1736.

Hibbard, J. H., E. R. Mahoney, J. Stockard and M. Tusler (2005). "Development and testing of a short form of the patient activation measure." Health Serv Res **40**(6 Pt 1): 1918-1930.

Lee, R. M., M. Draper and S. Lee (2001). "Social connectedness, dysfunctional interpersonal behaviors, and psychological distress: Testing a mediator model." Journal of Counseling Psychology **48**: 310-318.

Lorig, K., A. Stewart, P. Ritter, V. González and et al. (1996). Outcome measures for health education and other health care interventions. Thousand Oaks, CA, US, Sage Publications, Inc.

Shin, C., S. H. Lee, K. M. Han, H. K. Yoon and C. Han (2019). "Comparison of the Usefulness of the PHQ-8 and PHQ-9 for Screening for Major Depressive Disorder: Analysis of Psychiatric Outpatient Data." Psychiatry Investig **16**(4): 300-305.

Skolasky, R. L., A. F. Green, D. Scharfstein, C. Boult, L. Reider and S. T. Wegener (2011). "Psychometric properties of the patient activation measure among multimorbid older adults." Health Serv Res **46**(2): 457-478.

Smith, C. E., I. Alam, C. Tan, B. C. Keegan and A. L. Blanchard (2022). "The Impact of Governance Bots on Sense of Virtual Community: Development and Validation of the GOV-BOTs Scale." Proc. ACM Hum.-Comput. Interact. **6**(CSCW2): Article 462.

Spitzer, R. L., K. Kroenke, J. B. Williams and B. Löwe (2006). "A brief measure for assessing generalized anxiety disorder: the GAD-7." Arch Intern Med **166**(10): 1092-1097.

Tennant, R., L. Hiller, R. Fishwick, S. Platt, S. Joseph, S. Weich, J. Parkinson, J. Secker and S. Stewart-Brown (2007). "The Warwick-Edinburgh Mental Well-being Scale (WEMWBS): development and UK validation." Health Qual Life Outcomes **5**: 63.

Tusa, N., H. Kautiainen, P. Elfving, S. Sinikallio and P. Mäntyselkä (2020). "Relationship between patient activation measurement and self-rated health in patients with chronic diseases." BMC Fam Pract **21**(1): 225.

Ware, J. E., Jr. and C. D. Sherbourne (1992). "The MOS 36-item short-form health survey (SF-36). I. Conceptual framework and item selection." Med Care **30**(6): 473-483.

Zhou, L., J. Bao, I. M. A. Setiawan, A. Saptono and B. Parmanto (2019). "The mHealth App Usability Questionnaire (MAUQ): Development and Validation Study." JMIR Mhealth Uhealth **7**(4): e11500.
