# Supplementary material for: Online Peer Support for Long-Term Conditions: Protocol for a Feasibility Randomized Controlled Trial
Source: JMIR Res Protoc. 2025 Jul 23;14:e71513. doi: 10.2196/71513 (PMC12329384; doi:10.2196/71513)
Supplement: Multimedia Appendix 3 [file resprot_v14i1e71513_app3.docx]

# Multimedia Appendix 3.

**CONSENT FORM** 1: Online peer support in long-term conditions: A feasibility randomised controlled trial (Eligibility Screening)

**Please complete this form after you have read the Information Sheet and/or listened to an explanation about the research.**

| **Title of project:** Online peer support in long-term conditions: A feasibility randomised controlled trial (Eligibility Screening) |
| --- |
| Name of Chief Investigator: Professor Matthew Hotopf  **Ethical review reference number: This project had been reviewed by South Central - Oxford C Research Ethics Committee (IRAS ID 328175).**  **Version number: V1.1** |
| Please select each statement box below and sign the consent form at the bottom of this page. |
| 1. I confirm that I have read and understood the Information Sheet (v1.1) dated (20.12.2023) for the above project. I understand why the research is being done and any risks involved.  I have had the opportunity to consider the information and ask questions which have been answered to my satisfaction. |
| 1. I confirm that I have read and understood why the eligibility screenings are required before participating in the research. I have had the opportunity to consider this information and ask questions about the eligibility screening(s) which have been answered to my satisfaction. |
| 1. I agree voluntarily to completing the eligibility screening(s) as part of this project and understand that I can refuse to take part and can withdraw from the project at any time, without having to give a reason without my medical care or legal rights being affected. I agree that if I decide to withdraw, data collected up until the point of withdrawal will be retained by the research team. |
| 1. I understand that agreeing to complete the eligibility screenings does not guarantee that I will be able to take part in the research project. |
| 1. I understand my personal information will be processed for the purposes explained to me in the Information Sheet. I understand that such information will be handled under the terms of UK data protection law, including the UK General Data Protection Regulation (UK GDPR) and the Data Protection Act 2018. |
| 1. I understand that the personal information I provide in the eligibility screenings will be anonymously analysed. I understand that this will be analysed regardless of whether I am eligible to participate in the research project.   __________________________________________________________________________________   1. I agree to the data I enter as part of this project to be stored on and accessed via Qualtrics management software. |
| 1. I understand that relevant sections of my medical notes and data collected during the eligibility screen, may be looked at by individuals from King’s College London, from regulatory authorities or from the NHS Trust Organisation, where it is relevant to my taking part in this research or for monitoring and audit purposes. I give permission for these individuals to have access to my records. |
| 1. I understand that my de-identified data may be used to support other research in the future and may be shared anonymously with other researchers/third parties upon their request. I understand that all requests will be extensively reviewed before approval and data sharing by the KCL research team. |
| 1. I understand that confidentiality and anonymity will be maintained, and it will not be possible to identify me in any publication or research outputs. |
| 1. I agree that the research team may use my data for future research and understand that any such use of identifiable data would be reviewed and approved by a research ethics committee as required. If this happens, I understand that my data would not be identifiable in any report. |
| 1. I understand that there are no direct benefits to myself from completing the eligibility screening(s). |
| 1. I agree to the research team using my email address and mobile/telephone number to contact during the research period. I understand that these contact details will not be shared beyond the research team and the software developer team (BitJam). |
| 1. I understand that this form relates to me agreeing to the eligibility screening(s) and that I will be required to complete a separate consent form for the research study should I be eligible and wish to participate. |

| **The following clauses are OPTIONAL.** If you do wish to be contacted in the future, you will be asked to provide your email address. |
| --- |
| 1. **Optional.** I agree that the researcher may retain my contact details so that I may be contacted in the future by King’s College London researchers who would like to invite me to participate in future studies of a similar nature. |

- **Yes, I agree to the above optional statement.**
- **No, I do not agree.**

You have indicated that you would like to be contacted about potential future research opportunities. Please enter your email address below.

Please type out your full name in the box below.

Please virtually ‘sign’ this consent form

Please type out today's date (dd/mm/yyyy)

Would you like to receive a copy of this consent form? Please note that this will be automatically generated through Qualtrics directly to the email address you provide.

- Yes, please send a copy
- No

Please provide your e-mail address below. Your consent form will be sent to you immediately. Please check your spam folder if it does not appear in your inbox.

**CONSENT FORM** 2**:** Online peer support in long-term conditions: A feasibility randomised controlled trial

**Please complete this form after you have read the Information Sheet and/or listened to an explanation about the research.**

| **Title of project:** Online peer support in long-term conditions: A feasibility randomised controlled trial | |
| --- | --- |
| Name of Chief Investigator: Professor Matthew Hotopf |  |
| Please select each statement box below and sign the consent form at the bottom of this page. | |
| 1. I confirm that I have read and understood the Information Sheet (v1.1) dated (20.12.2023) for the above project. I understand why the research is being done and any potential risks involved. I have had the opportunity to consider the information and ask questions, which have been answered to my satisfaction. | |
| 1. I agree voluntarily to be a participant in this project and understand that I can refuse to take part and can withdraw from the project at any time, without having to give a reason without my medical care or legal rights being affected. I agree that if I decide to withdraw, data collected up until the point of withdrawal will be retained by the research team. | |
| 1. I understand my personal information will be processed for the purposes explained to me in the Information Sheet. I understand that such information will be handled under the terms of UK data protection law, including the UK General Data Protection Regulation (UK GDPR) and the Data Protection Act 2018. | |
| 1. I understand that relevant sections of my medical notes and data collected during the study, may be looked at by individuals from King’s College London, from regulatory authorities or from the NHS Trust Organisation, where it is relevant to my taking part in this research or for monitoring and audit purposes. I give permission for these individuals to have access to my records. | |
| 1. I understand that confidentiality and anonymity will be maintained, and it will not be possible to identify me in any publication or research outputs. | |
| 1. I understand that if I participate in the online peer support intervention, “CommonGround”, that I am responsible for maintaining my own and other users’ anonymity and confidentiality.  I understand that I am responsible for anything that I post or share on the platform. | |
| 1. I understand that to participate in the online peer support platform I will need to agree to the Community Principles (‘terms of use’) to gain access to the platform, and that if I breach these terms, the research or moderation team may contact me privately (i.e., link my anonymous username to my personal information). | |
| 1. I agree to my data being shared with third parties (BitJam (software developers) and a transcription service (if applicable)) that are within the UK as outlined in the Information Sheet. I also agree to the data I enter as part of this project to be stored on and accessed via Qualtrics management software. | |
| 1. I agree that the research team may use my data for future research and understand that any such use of identifiable data would be reviewed and approved by a research ethics committee as required. If this happens, I understand that my data would not be identifiable in any report. | |
| 1. I understand that my de-identified data may be used to support other research in the future and may be shared anonymously with other researchers/third parties upon their request. I understand that all requests will be extensively reviewed before approval and data sharing by the KCL research team. | |
| 1. If selected for the interview stage of the research, I agree to my participation in the interview being audio recorded and/or video recorded (if via online video chat) and to the publication of direct de-identified quotes. I understand that I can chose not to participate in any interview without it impacting my participation in the rest of the study. I also agree to my original interview audio file being shared with a transcription service, who will transcribe my interview and share with KCL. I understand that the transcription service will not retain the original interview recording. | |
| 1. I understand that I cannot take part if I fall under the exclusion criteria as detailed in the information sheet. | |
| 1. I understand that if I disclose information indicating that there is a safety (myself or others) or crime-related concern, the research team and/or the moderation team may contact me privately and/or be obliged to break anonymity and confidentiality to notify appropriate services, in accordance with our Moderation and Safeguarding policies. I understand that I will be notified of this if this is the case. | |
| 1. I understand that the “CommonGround” platform is not a crisis support service, cannot provide one-to-one support or advice, and will not be monitored by the research team 24/7. | |
| 1. I understand that there might not be any direct benefits to myself from participating in the study, and that I might not be given access to the online peer support platform. | |
| 1. I understand the potential risks of participating in this study, as described in the information sheet. | |
| 1. I understand that participating in this research will not affect the medical care that I receive and that I can continue my usual care throughout the trial. | |
| 1. I agree to the research team using my email address and mobile/telephone number to contact me during the research period. I understand that these contact details will not be shared beyond the research team (King’s College London) and the software developer team (BitJam). | |
| 1. I agree to taking part in this study. | |

Please type out your full name in the box below to ‘sign’ this consent form. Please check that you have selected all nineteen items above if you wish to participate in our research.

Please virtually ‘sign’ this consent form

Please enter today's date (dd/mm/yyyy)

Would you like to receive a copy of this consent form? Please note that the research team will send this to you directly via e-mail as soon as possible.

- Yes, send me a copy
- No

Please enter your email address so that the research team can send you your consent form.
