# Supplementary material for: Online Peer Support for Long-Term Conditions: Protocol for a Feasibility Randomized Controlled Trial
Source: JMIR Res Protoc. 2025 Jul 23;14:e71513. doi: 10.2196/71513 (PMC12329384; doi:10.2196/71513)

# Multimedia Appendix 4.

Screenshot 1. The landing page of CommonGround


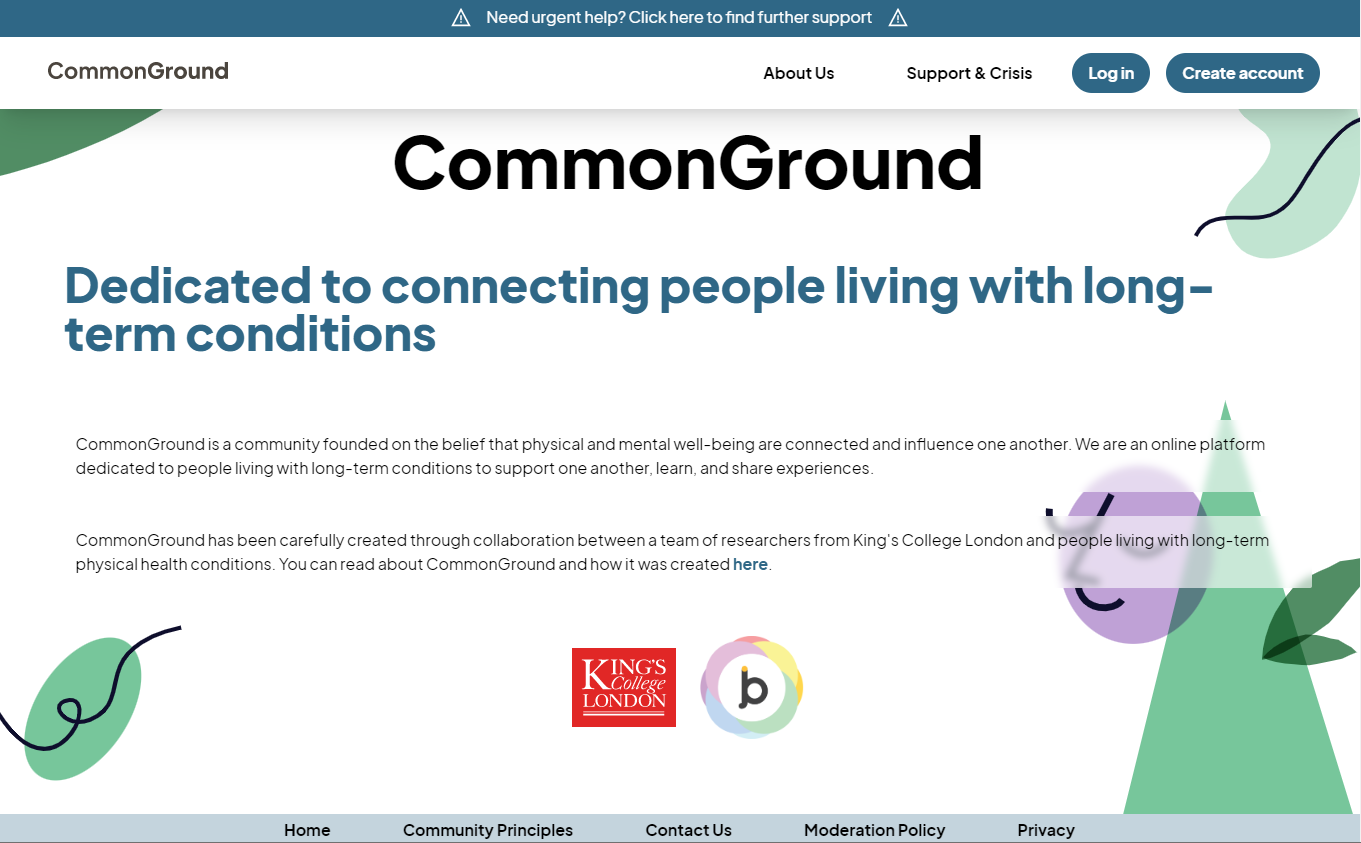


Screenshot 2. The community feed of CommonGround (peer support component)


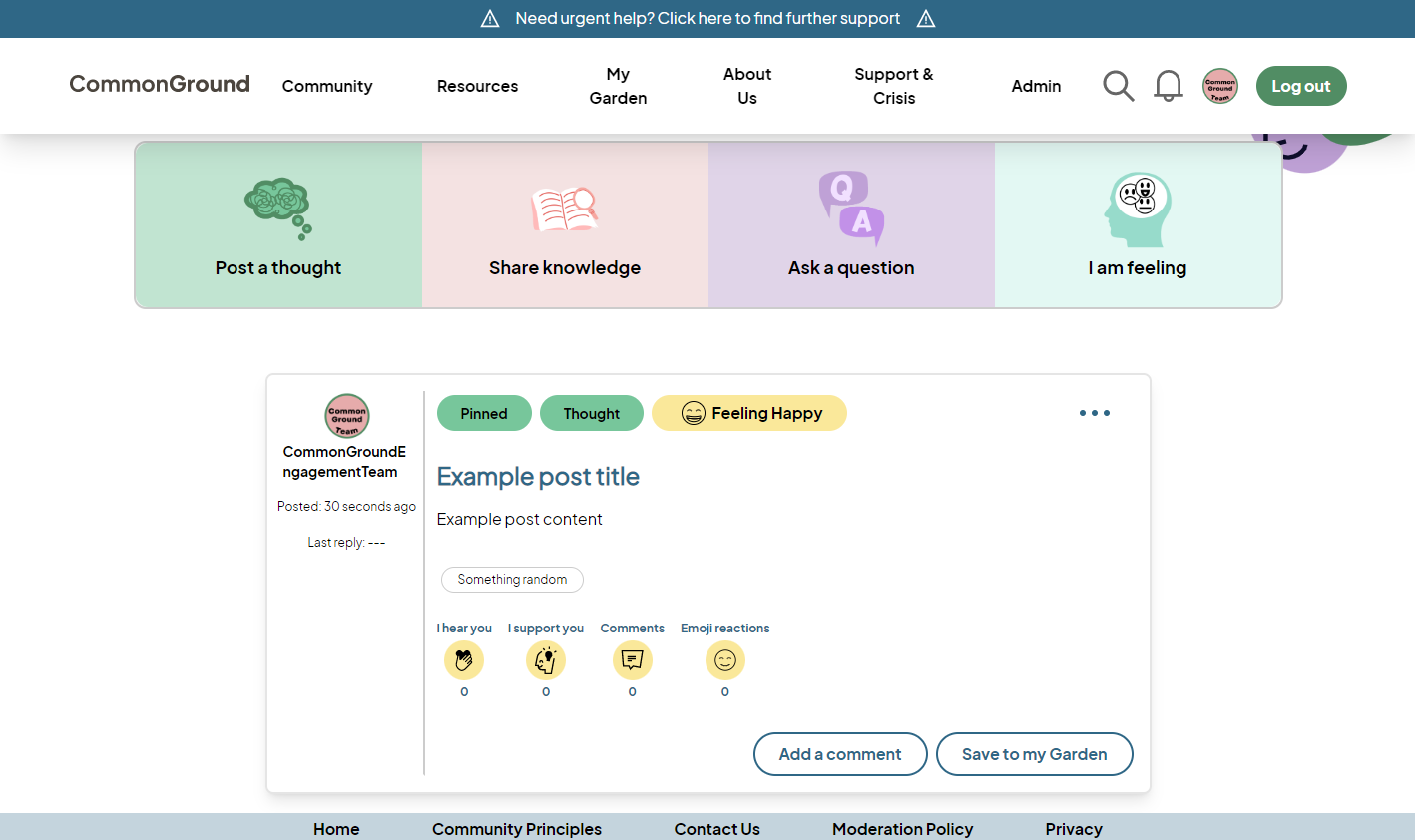


Screenshot 3. The resources page of CommonGround (psychoeducation component)


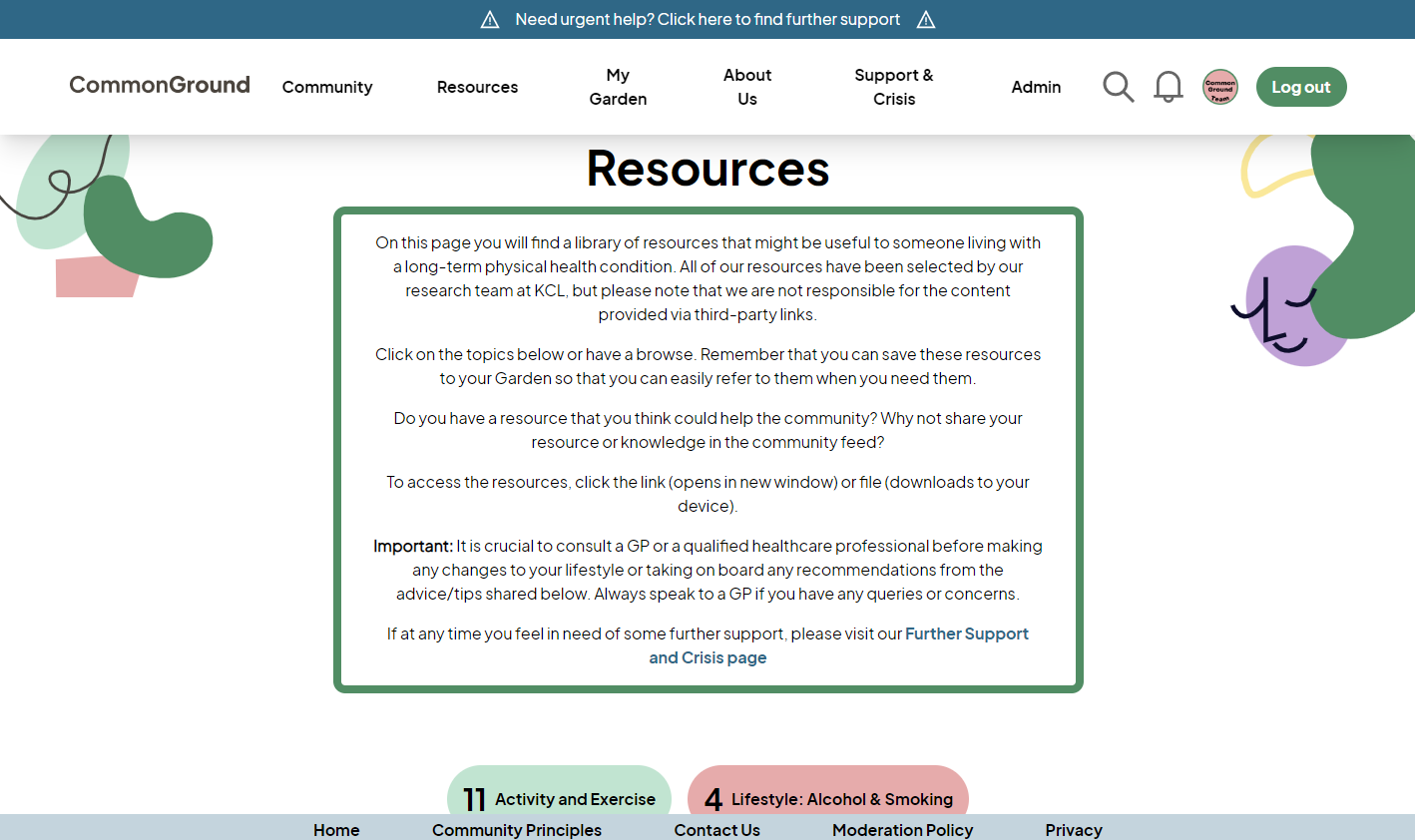


Screenshot 4. The ‘My Garden’ page of CommonGround


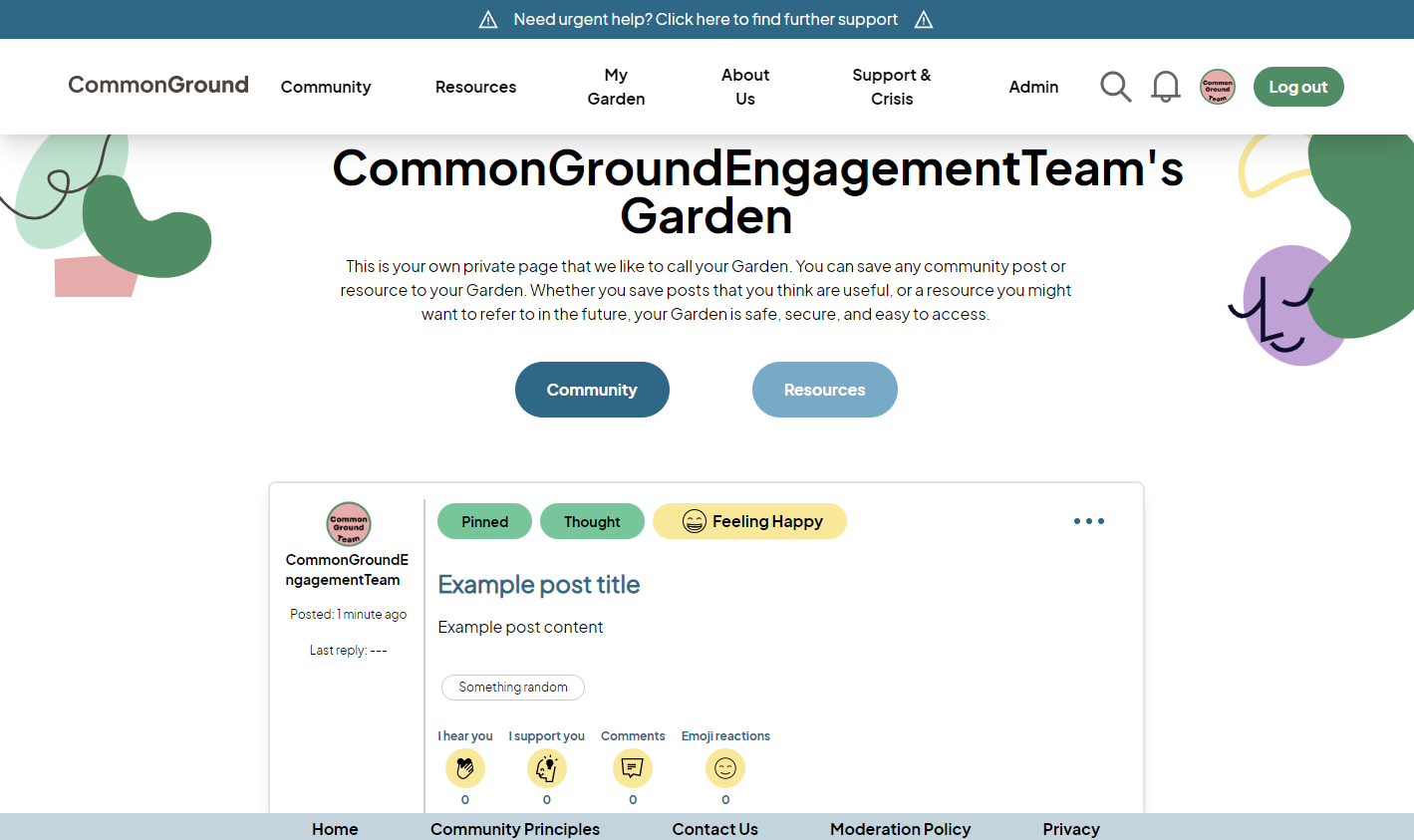


Screenshot 5. Search box and search results page of CommonGround


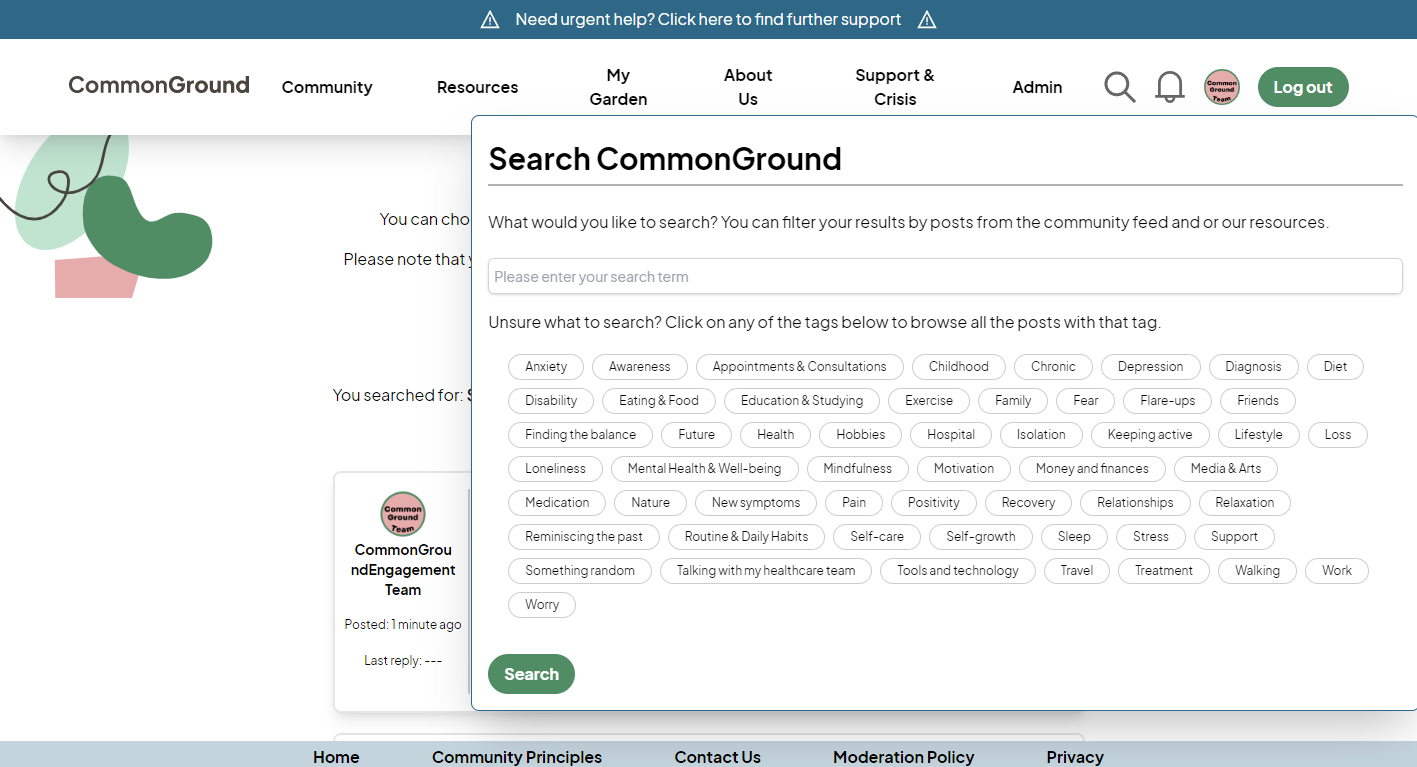


Screenshot 6. Further support and crisis page


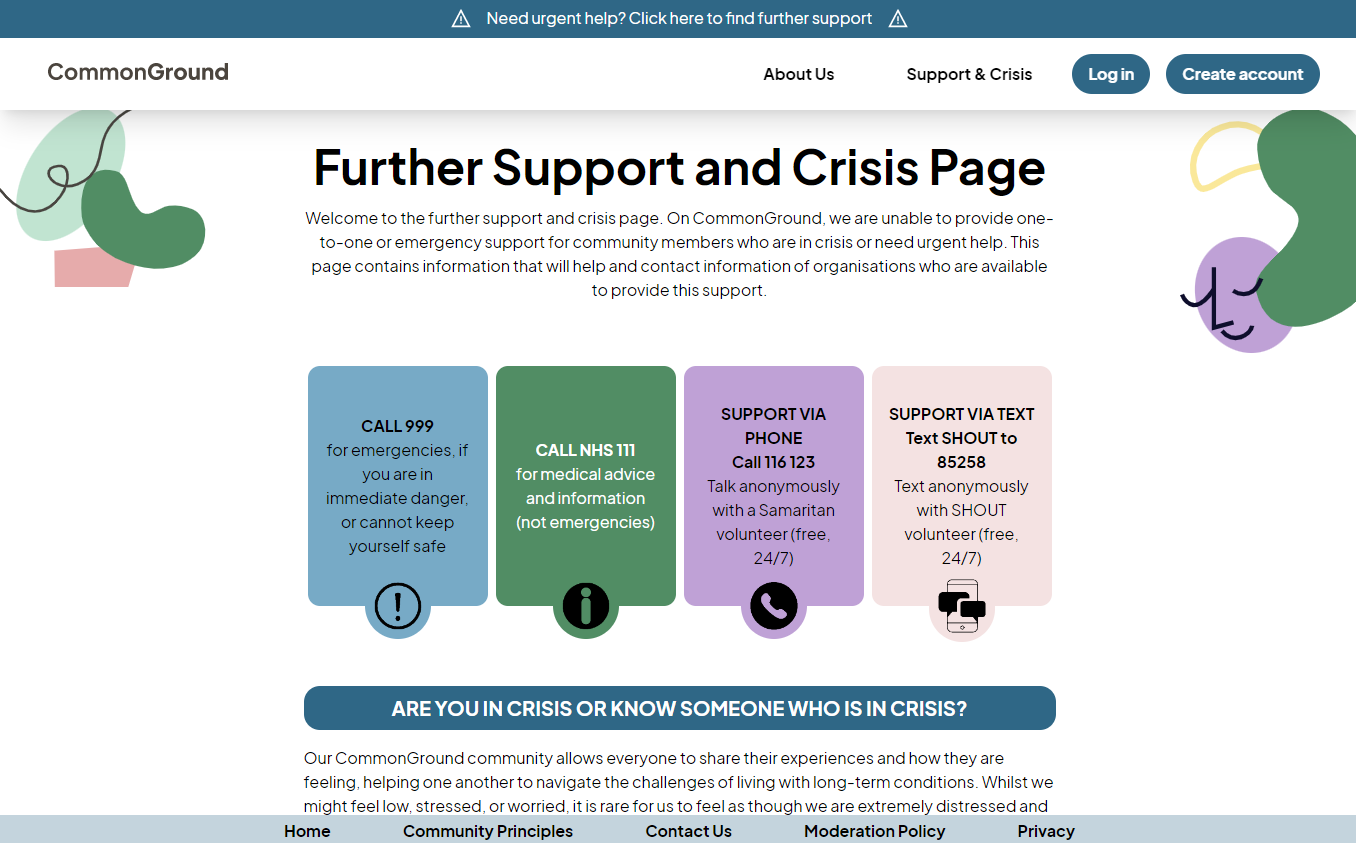


Screenshot 7. Moderation policy page


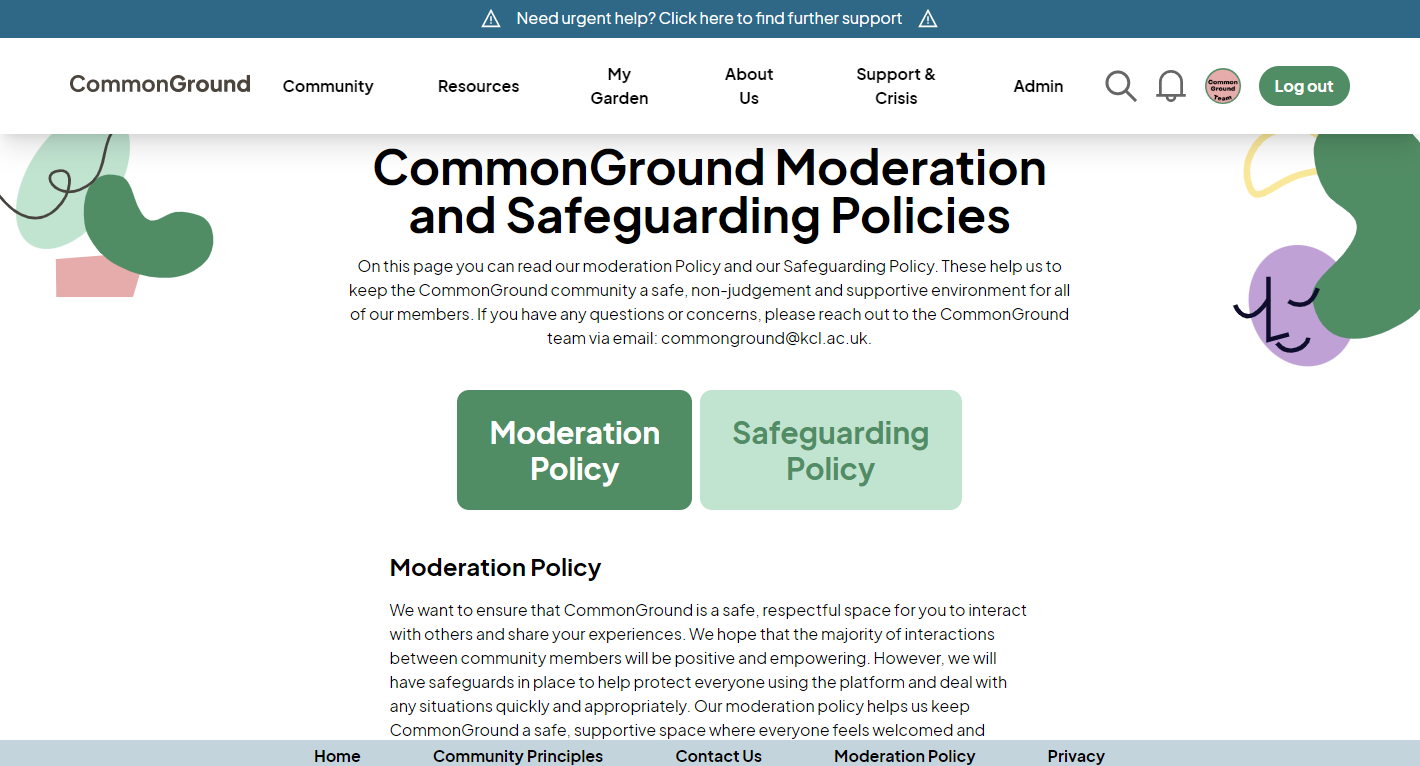


Screenshot 8. Admin panel


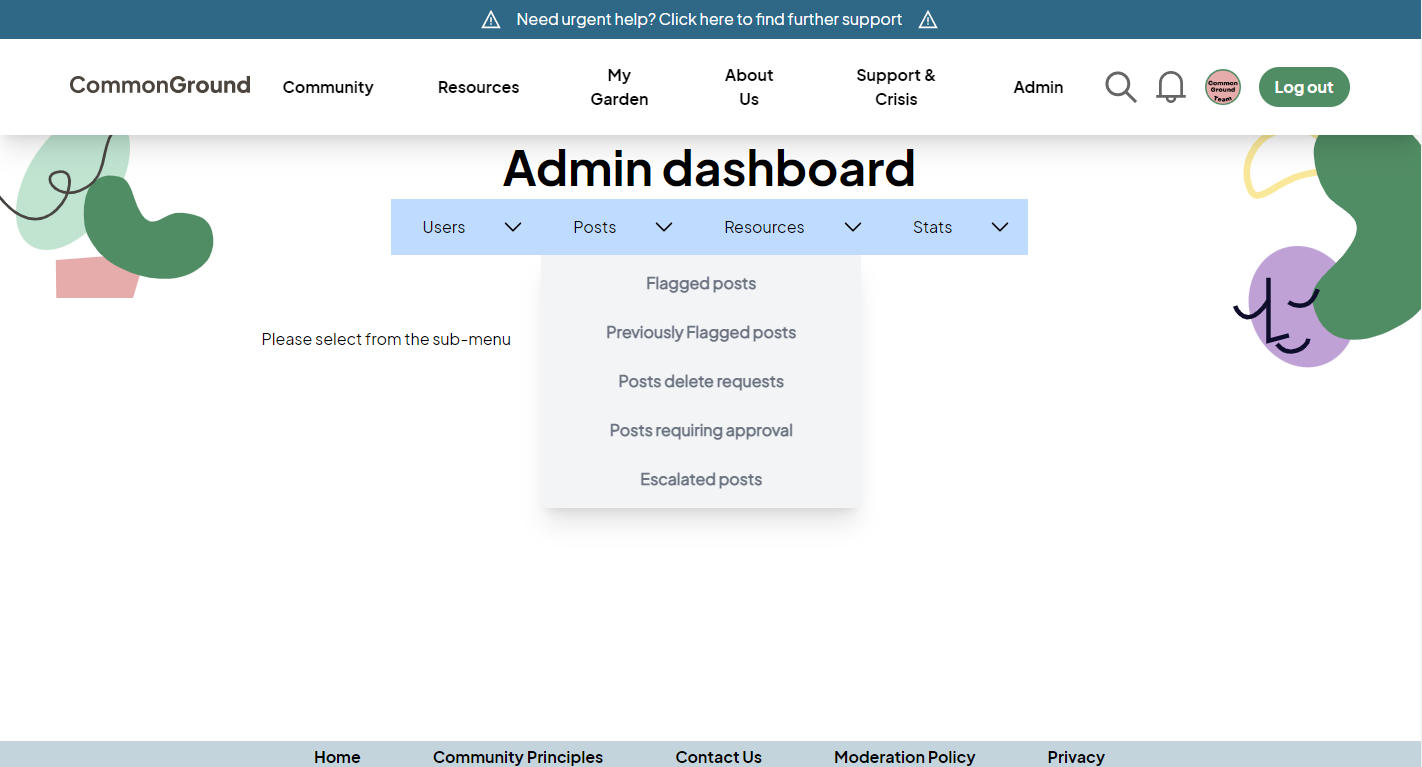

Supplement: Multimedia Appendix 4 [file resprot_v14i1e71513_app4.docx]
